# Generalizing Bayesian phylogenetics to infer shared evolutionary events

**DOI:** 10.1073/pnas.2121036119

**Published:** 2022-07-15

**Authors:** Jamie R. Oaks, Perry L. Wood, Cameron D. Siler, Rafe M. Brown

**Affiliations:** ^a^Department of Biological Sciences, Auburn University, Auburn, AL 36849;; ^b^Museum of Natural History, Auburn University, Auburn, AL 36849;; ^c^Sam Noble Oklahoma Museum of Natural History, University of Oklahoma, Norman, OK 73072-7029;; ^d^Department of Biology, University of Oklahoma, Norman, OK 73019;; ^e^Biodiversity Institute, University of Kansas, Lawrence, KS 66045;; ^f^Department of Ecology and Evolutionary Biology, University of Kansas, Lawrence, KS 66045

**Keywords:** phylogenetics, Bayesian, shared divergence, multifurcation, Gekkonidae

## Abstract

Many processes of biological diversification can simultaneously affect multiple evolutionary lineages. Examples include multiple members of a gene family diverging when a region of a chromosome is duplicated, multiple viral strains diverging at a “super-spreading” event, and a geological event fragmenting whole communities of species. It is difficult to test for patterns of shared divergences predicted by such processes because all phylogenetic methods assume that lineages diverge independently. We introduce a Bayesian phylogenetic approach to relax the assumption of independent, bifurcating divergences by expanding the space of topologies to include trees with shared and multifurcating divergences. This allows us to jointly infer phylogenetic relationships, divergence times, and patterns of divergences predicted by processes of diversification that affect multiple evolutionary lineages simultaneously or lead to more than two descendant lineages. Using simulations, we find that the method accurately infers shared and multifurcating divergence events when they occur and performs as well as current phylogenetic methods when divergences are independent and bifurcating. We apply our approach to genomic data from two genera of geckos from across the Philippines to test if past changes to the islands’ landscape caused bursts of speciation. Unlike previous analyses restricted to only pairs of gecko populations, we find evidence for patterns of shared divergences. By generalizing the space of phylogenetic trees in a way that is independent from the likelihood model, our approach opens many avenues for future research into processes of diversification across the life sciences.

## Significance

Phylogenetic models have long assumed that lineages diverge independently. Processes of diversification that are of interest in biogeography, epidemiology, and genome evolution violate this assumption by affecting multiple evolutionary lineages. To relax the assumption of independent divergences and infer patterns of divergences predicted by such processes, we introduce a way of conceptualizing, modeling, and inferring phylogenetic trees. We apply the approach to genomic data from geckos distributed across the Philippines and find support for patterns of shared divergences predicted by repeated fragmentation of the archipelago by interglacial rises in sea level.

There are many processes of biological diversification that affect multiple evolutionary lineages, generating patterns of temporally clustered divergences across the tree of life. Understanding such processes of diversification has important implications across many fields and scales of biology. At the scale of genome evolution, the duplication of a chromosome segment harboring multiple members of a gene family causes multiple, simultaneous (or “shared”) divergences across the phylogenetic history of the gene family (1–4). In epidemiology, when a pathogen is spread by multiple infected individuals at a social gathering, this will create shared divergences across the pathogen’s “transmission tree” (5–7). If one of these individuals infects two or more others, this will create a multifurcation (a lineage diverging into three or more descendants) in the transmission tree. At regional or global scales, when biogeographic processes fragment communities, this can cause shared divergences across multiple affected species (8–13). If the landscape is fragmented into three or more regions, this can also cause multifurcations (14). For example, the repeated fragmentation of the Philippines by interglacial rises in sea level since the late Pliocene (15–19) has been an important model to help explain remarkably high levels of microendemism and biodiversity across the archipelago (20–30). This model predicts that recently diverged taxa across the islands should have (potentially multifurcating) divergence times clustered around the beginning of interglacial periods. We are limited in our ability to infer patterns of divergences predicted by such processes because phylogenetic methods assume that lineages diverge independently.

**Fig. 1.**
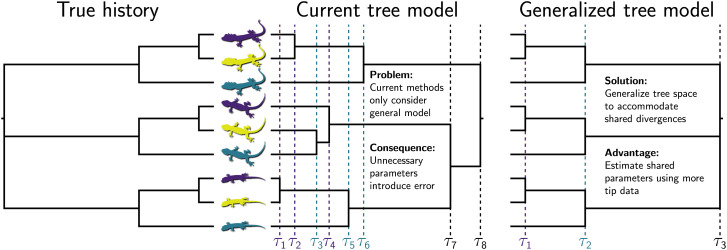
A hypothetical evolutionary history with shared divergences (*Left*) and the benefits of the generalizing tree space under such conditions (*Right*). Current methods are restricted to one class of tree models, where the tree is fully bifurcating and independent divergence-time parameters are estimated for all internal nodes (*Center*). Figure was made by using Gram [v4.0.0 (35)] and the P4 phylogenetic toolkit [v1.4 5742542 (36)]. Image credit for top and bottom lizard silhouettes: Phylopic/Steven Traver. Image credit for middle lizard silhouettes: Pixabay/No-longer-here.

To formalize this assumption of independent divergences and develop ways to relax it, it is instructive to view phylogenetic inference as an exercise of statistical model selection, where each topology is a separate model (31–33). Current methods for estimating rooted phylogenies with *N* tips only consider tree models with N−1 bifurcating divergences and assume that these divergences are independent, conditional on the topology (see ref. 34 for multifurcations in unrooted trees). If, in the history leading to the tips we are studying, diversification processes affected multiple lineages simultaneously or caused them to diverge into more than two descendants, the true tree could have shared or multifurcating divergences. This would make current phylogenetic models with N−1 independent divergence times overparameterized, introducing unnecessary error (Fig. 1). Even worse, with current methods, we lack an obvious way of using our data to test for patterns of shared or multifurcating divergences predicted by such processes.

We relax the assumption of independent, bifurcating divergences by introducing a Bayesian approach to generalizing the space of tree models to allow for shared and multifurcating divergences. In our approach, we view trees with N−1 bifurcating divergences as only one class of tree models in a greater space of trees with anywhere from 1 to N−1 potentially shared and/or multifurcating divergences (*SI Appendix*, Fig. S1). We introduce reversible-jump Markov chain Monte Carlo (MCMC) algorithms (37–39) to sample this generalized space of trees, allowing us to jointly infer evolutionary relationships, shared and multifurcating divergences, and divergence times. We couple these algorithms with a likelihood model for directly calculating the probability of biallelic characters, given a population (or species) phylogeny, while analytically integrating over all possible gene trees under a coalescent model and all possible mutational histories under a finite-sites model of character evolution (40, 41). Using simulations, we find that the generalized tree model accurately infers shared and multifurcating divergences, while maintaining a low rate of falsely inferring such divergences. To test for patterns of shared and multifurcating divergences predicted by repeated fragmentation of the Philippines by interglacial rises in sea level (42–44), we apply the generalized tree model to genomic data from two genera of geckos codistributed across the islands.

## Results

### Simulations on Fixed Trees.

**Fig. 2.**
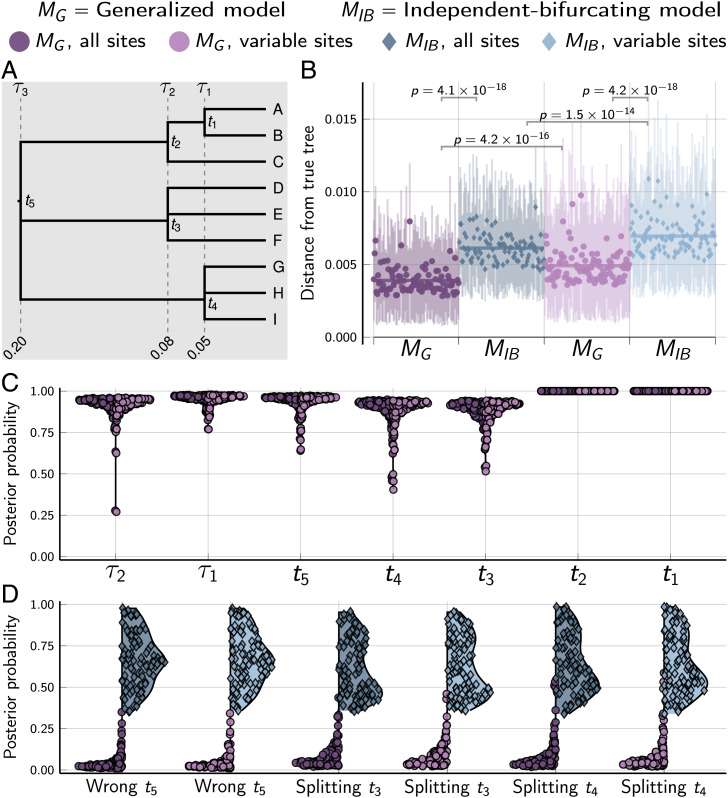
Results of analyses of 100 datasets, each with 50,000 biallelic characters simulated on the species tree shown in *A* with divergence times in units of expected substitutions per site. (*B*) The square root of the sum of squared differences in branch lengths between the true tree and each posterior tree sample (46); the point and bars represent the posterior mean and equal-tailed 95% credible interval, respectively. *P* values are shown for Wilcoxon signed-rank tests (47) comparing the paired differences in tree distances between methods. (*C*) Violin plots of the posterior probabilities of each node and shared divergence in the true tree across the 100 simulated datasets. (*D*) Violin plots of the most probable incorrect root node and most probable of the three incorrect splittings of the *t*_3_ and *t*_4_ multifurcations. For each simulation, the mutation-scaled effective population size ($N_{e}\mu$) was drawn from a gamma distribution (shape = 20, mean = 0.001) and shared across all the branches of the tree; this distribution was used as the prior in analyses. Tree was plotted by using Gram [v4.0.0, Commit 02286362 (35)] and the P4 phylogenetic toolkit [v1.4, Commit d9c8d1b1 (36)]. Other plots were created by using the PGFPlotsX [v1.2.10, Commit 1adde3d0 (48)] backend of the Plots [v1.5.7, Commit f80ce6a2 (49)] package in Julia [v1.5.4 (50)].

The generalized tree model (*M_G_*) sampled trees significantly closer (45, 46) to the true tree than an otherwise-equivalent model that assumes independent, bifurcating divergences (*M_IB_*), when applied to 100 datasets simulated along the species tree in Fig. 2*A*, each with 50,000 unlinked biallelic characters (Fig. 2*B*). From these simulated data, the generalized model consistently inferred the correct shared and multifurcating divergences with high posterior probabilities (Fig. 2*C*). Unlike the independent-bifurcating model, the generalized approach avoids strong support for nonexistent branches that spuriously split truly multifurcating nodes (Fig. 2*D*). Under both models, analyzing only the variable characters causes a reduction in tree accuracy (Fig. 2*B*), but yields similar posterior probabilities for shared and multifurcating divergences (Fig. 2*C*).

**Fig. 3.**
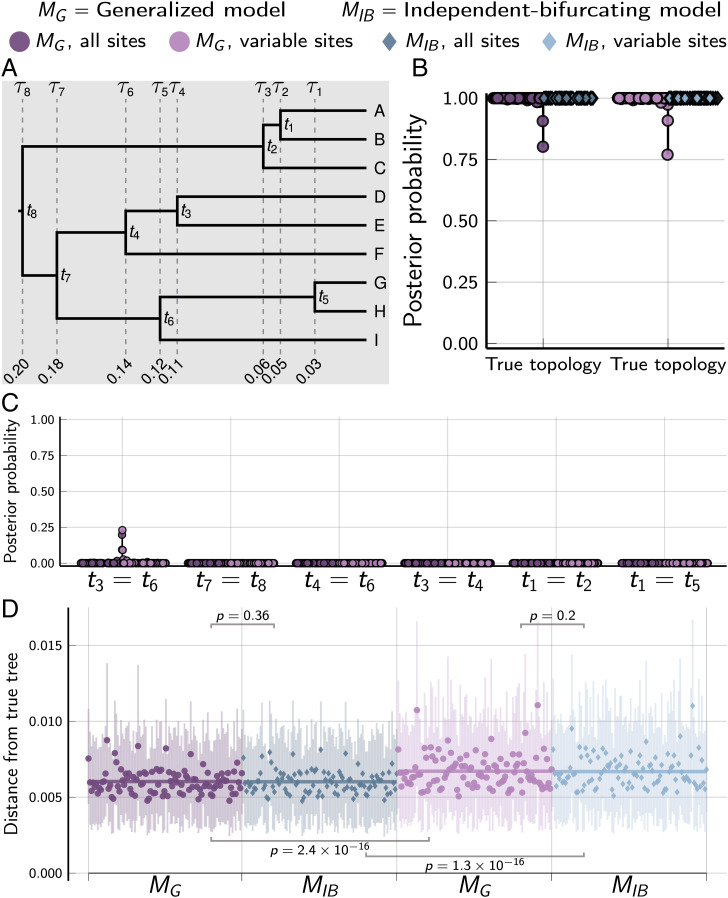
Results of analyses of 100 datasets, each with 50,000 biallelic characters simulated on the species tree shown in *A* with divergence times in units of expected substitutions per site. (*B*) The posterior probability of the true topology. (*C*) The posterior probability of incorrectly shared or multifurcating nodes. (*D*) The square root of the sum of squared differences in branch lengths between the true tree and each posterior tree sample (46); the point and bars represent the posterior mean and equal-tailed 95% credible interval, respectively. *P* values are shown for Wilcoxon signed-rank tests (47) comparing the paired differences in tree distances between methods. For each simulation, the mutation-scaled effective population size ($N_{e}\mu$) was drawn from a gamma distribution (shape = 20, mean = 0.001) and shared across all the branches of the tree; this distribution was used as the prior in analyses. Tree was plotted by using Gram [v4.0.0, Commit 02286362 (35)] and the P4 phylogenetic toolkit [v1.4, Commit d9c8d1b1 (36)]. Other plots were created by using the PGFPlotsX [v1.2.10, Commit 1adde3d0 (48)] backend of the Plots [v1.5.7, Commit f80ce6a2 (49)] package in Julia [v1.5.4 (50)].

When applied to datasets of 50,000 characters simulated along a tree with independent, bifurcating divergences (Fig. 3*A*), both the *M_G_* and *M_IB_* models consistently inferred the correct topology with strong support (Fig. 2*B*), and the *M_G_* method did not support incorrect shared or multifurcating divergences (Fig. 3*C*). This was true whether all the characters or only the variable characters were analyzed (Fig. 3 *B* and *C*). Looking at the distances (45, 46) between the trees from the posterior samples and the true tree, there is no difference between the *M_G_* and *M_IB_* models when the true tree has only independent, bifurcating divergences (Fig. 3*D*). For both models, using all the characters yields posterior samples of more accurate trees than only analyzing variable characters (Fig. 3*D*).

### Simulations on Random Trees.

**Fig. 4.**
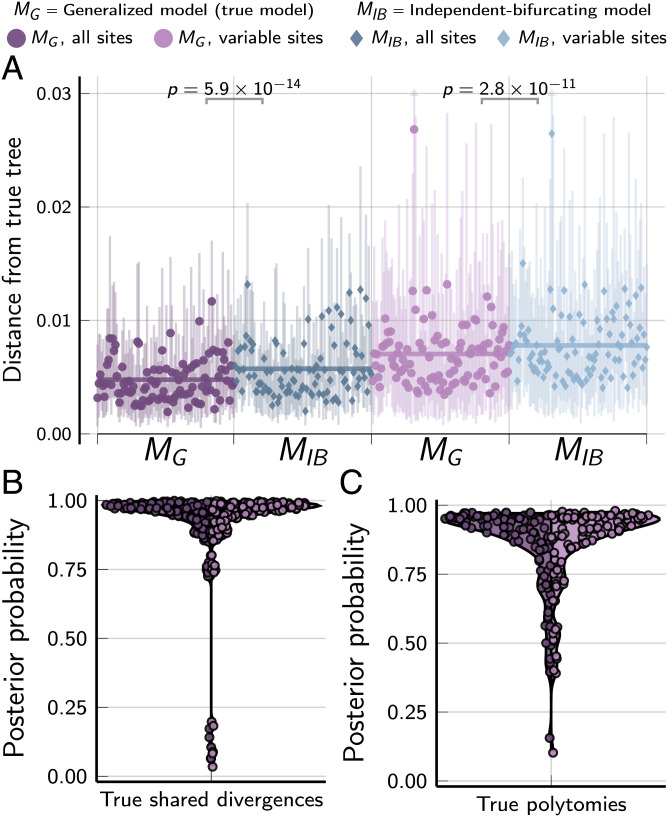
The performance of the *M_G_* and *M_IB_* tree models when applied to 100 datasets, each with 50,000 biallelic characters simulated on species trees randomly drawn from the *M_G_* tree distribution. (*A*) The square root of the sum of squared differences in branch lengths between the true tree and each posterior tree sample (46); the point and bars represent the posterior mean and equal-tailed 95% credible interval, respectively. *P* values are shown for Wilcoxon signed-rank tests (47) comparing the paired differences in tree distances between methods. Violin plots show posterior probabilities of all true shared divergences (*B*) and multifurcating nodes (*C*) across all simulated trees. For each simulation, the mutation-scaled effective population size ($N_{e}\mu$) was drawn from a gamma distribution (shape = 20, mean = 0.001) and shared across all the branches of the tree; this distribution was used as the prior in analyses. Plots were created by using the PGFPlotsX [v1.2.10, Commit 1adde3d0 (48)] backend of the Plots [v1.5.7, Commit f80ce6a2 (49)] package in Julia [v1.5.4 (50)].

When we simulated 100 datasets (each with nine species and 50,000 characters), where the true tree and divergence times were randomly drawn from the generalized tree distribution (*M_G_*), we again found that the *M_G_* performs better than the *M_IB_* at inferring the correct tree and divergence times (Fig. 4*A*) and generally recovers true shared and multifurcating divergences with moderate to strong support (Fig. 4 *B* and *C*). When the tree and divergence times were randomly drawn from an independent, bifurcating tree model (*M_IB_*), the generalized model performs similarly to the true model (*SI Appendix*, Fig. S2).

Both the *M_G_* and *M_IB_* models accurately and precisely estimate the age of the root, tree length, and effective population size from the datasets simulated on random *M_G_* and *M_IB_* trees (top two rows of *SI Appendix*, Figs. S3–S5, respectively). Accuracy is similar with and without constant characters, but precision is higher when including constant characters.

### The Rate of Falsely Inferring Shared Divergences.

**Fig. 5.**
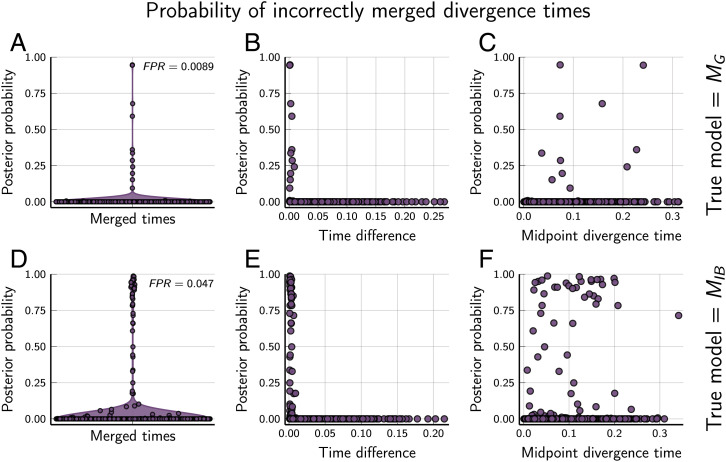
The *M_G_* tree model has a low false-positive rate [FPR; the proportion of incorrectly merged divergence times (*A* and *D*) with a posterior probability > 0.5] when applied to data simulated on trees drawn from the *M_G_* (*A*–*C*) and *M_IB_* (*D*–*F*) models. Support for incorrectly merged divergence times is high only when the difference between the times is small (*B* and *E*) and is not correlated with the age of the merged nodes; *P* = 0.11 (*C*) and 0.25 (*F*) for a *t* test in which Pearson’s correlation coefficient = 0 using all points with posterior probability > 0. Time units are expected substitutions per site. Plots were created by using the PGFPlotsX [v1.2.10, Commit 1adde3d0 (48)] backend of the Plots [v1.5.7, Commit f80ce6a2 (49)] package in Julia [v1.5.4 (50)].

To quantify the rate at which phycoeval incorrectly infers shared and/or multifurcating divergences, we used the results from the *M_G_* analyses of the datasets simulated on random trees from the *M_G_* and *M_IB_* models. From the posterior sample of each analysis, we used sumphycoeval to calculate the proportion of samples that contained incorrectly merged neighboring divergence times. To do this, we merged all possible neighboring divergence times from the true tree, each of which creates a shared divergence or multifurcation, and counted how many posterior samples contained each divergence scenario. We found that phycoeval had a low false-positive rate for the simulated data; less than 1% (Fig. 5*A* and *SI Appendix*, Fig. S6) and 5% (Fig. 5*D* and *SI Appendix*, Fig. S7) of incorrectly merged divergence times had an approximate posterior probability greater than 0.5 when analyzing data simulated on trees sampled from the *M_G_* and *M_IB_* models, respectively. In all cases with moderate to strong support for falsely merged divergences, the difference in time between the merged divergences was small (<0.005 expected substitutions per site; Fig. 5 *B* and *E*). There was no correlation between support for incorrectly merged divergences and their age (Fig. 5 *C* and *F*; the *P* value for a *t* test that Pearson’s correlation coefficient = 0 using all points with posterior probability >0 was 0.11 and 0.25 for results from data simulated under *M_G_* and *M_IB_*, respectively).

### Convergence and Mixing of MCMC Chains.

For all analyses of simulated data, the root age, tree length, and effective population size had a potential-scale reduction factor (PSRF; the square root of equation 1.1 in ref. 51) less than 1.2 and an effective sample size [ESS (52)] greater than 200. The average SD of split frequencies (ASDSF) among the four MCMC chains was less than 0.017 for all analyses and less than 0.01 for most (*SI Appendix*, Fig. S8).

Convergence and mixing were better under *M_G_* than *M_IB_* when applied to datasets simulated on trees with shared or multifurcating divergences (*SI Appendix*, Fig. S8, *Left*). When applied to datasets simulated with no shared or multifurcating divergences, MCMC performance was similar between *M_G_* and *M_IB_* (*SI Appendix*, Fig. S8, *Right*).

The MCMC settings used for *M_G_* and *M_IB_* are identical except for the reversible-jump moves that add or remove divergence-time parameters are turned off under the latter model. Under the *M_IB_* model, the tree topology is updated by several MCMC moves (*SI Appendix*, sections 4.5 and 4.6.1), which performed well when divergences were independent and bifurcating (Fig. 3 and *SI Appendix*, Fig. S8). To further probe the improved MCMC behavior of *M_G_* in the face of shared and multifurcating divergences, we reran the analyses under the *M_IB_* model on the 100 datasets simulated along the tree in Fig. 2*A* with more favorable MCMC settings. In these *M_IB_* reanalyses, we ran the MCMC chains twice as long, sampled them half as frequently, and started them with the correct tree. The results were nearly identical to the original MCMC chains under the *M_IB_* model (*SI Appendix*, Fig. S9), suggesting that the improved mixing under *M_G_* was not simply due to insufficient MCMC sampling effort under the *M_IB_* model.

### Simulations of Linked Characters.

The multispecies coalescent likelihood we have coupled with our generalized tree model assumes that each biallelic character is unlinked [i.e., each character evolved along a gene tree that was independent of other characters, conditional on the species tree (40, 41)]. However, each locus comprising the gecko datasets we analyzed (see below) consists of ∼90 contiguous nucleotides. To assess whether linked sites might bias our results, we repeated the simulations above, but with 500 loci, each with 100 linked characters. When all characters (variable and constant) were analyzed, results from the datasets simulated with linked characters were very similar to results from unlinked characters above (*SI Appendix*, Figs. S3–S7 and S10–S13). When all but one variable character per locus was discarded to avoid violating the assumption of unlinked characters, performance was greatly reduced due to the large loss of data (*SI Appendix*, Figs. S3–S7 and S10–S13). These results suggest that the model is robust to linked characters, and it is better to analyze all sites from multilocus datasets, rather than reduce them to only one single-nucleotide polymorphism (SNP) per locus.

### Testing for Shared Divergences in Philippine Gekkonids Predicted by Glacial Cycles.

If the repeated fragmentation of the Philippines by interglacial rises in sea level generated pulses of speciation, taxa distributed across the archipelago should have divergence times clustered around the beginning of interglacial periods. We tested this prediction by applying our generalized tree model to restriction site-associated DNA sequencing (RADseq) data from species of *Cyrtodactylus* and *Gekko* collected from 27 and 26 locations across the islands, respectively (*SI Appendix*, Tables S1 and S2). We analyzed each genus separately because the rate of mutation differs between the genera, and phycoeval currently assumes a strict clock (though this is not required by the generalized tree model).

**Fig. 6.**
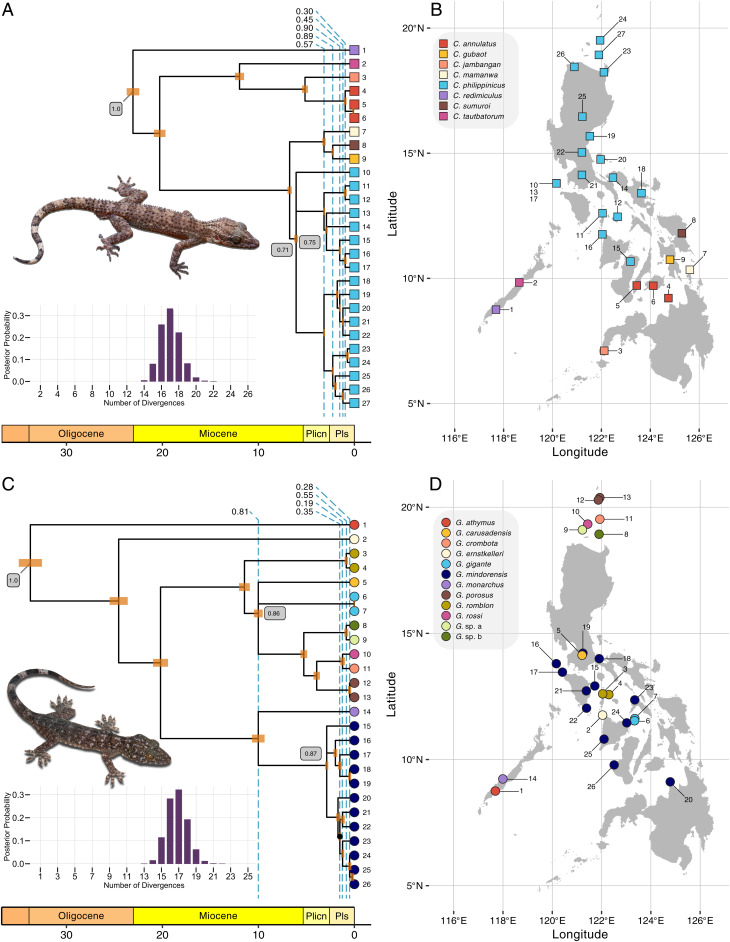
A summary of the generalized trees inferred from the *Cyrtodactylus* (*A* and *B*) and *Gekko* RADseq (*C* and *D*) datasets. The MAP tree is shown for both genera, along with the approximate posterior probabilities of the number of divergences. Shared divergences in MAP trees are indicated by dashed lines, with approximate posterior probabilities shown along the top. All clades (splits) had approximate posterior probabilities (PP) greater than 0.95, except for one indicated with a dot (PP = 0.89) within *G. mindorensis* (*C*). Approximate posterior probabilities of nodes are shown in gray boxes for the root and multifurcating nodes. To illustrate timescale, branch lengths of posterior samples of trees were rescaled from expected substitutions per site to millions of years using secondary calibrations (*Materials and Methods*). The top photo of *Cyrtodactylus* sp. is by C.D.S.; the bottom photo of *Gekko* sp. is by Jason Fernandez and R.M.B. Created using ggplot2 [v3.3.5 (53)], ggtree [v3.1.0 (54)], treeio [v1.17.0 (55)], deeptime [v0.0.6 (56)], cowplot [v1.1.1 (57)], and ggrepel [v0.9.1 (58)]. Links to nexus-formatted annotated trees: *Cyrtodactylus* and *Gekko*. Plicn, Pliocene; Pls, Pleistocene.

The maximum a posteriori (MAP) trees for both genera had 16 divergence times and weak to moderate support for five shared divergences (Fig. 6; see *SI Appendix*, Figs. S14 and S15 and Table S3 for more details about shared divergences). The MAP tree of *Cyrtodactylus* and *Gekko* had three and two multifurcations, respectively. For both genera, two of the shared divergences involved three nodes, and of the remaining three that involved two nodes, one involved a trichotomous node (three descending lineages). There were no other strongly supported shared divergences that were not included in the MAP trees of either genera. Most of the shared and multifurcating divergences occurred after the late Pliocene (Fig. 6 and *SI Appendix*, Table S3), based on rescaling the branch lengths of the posterior sample of trees from expected substitutions per site to millions of years using secondary calibrations (*Materials and Methods*).

For both genera, the number of divergence times with the highest approximate posterior probability (0.33 for *Cyrtodactylus* and 0.32 for *Gekko*) was 17, and the 95% credible interval spanned 15 to 19 divergences (Fig. 6). No trees with more than 22 divergence times were sampled for either genera, making the approximate posterior probability of 23 or more divergences less than $2.9\times 10^{-5}$ for both genera. The ASDSF (0.0027 for *Cyrtodactylus* and 0.0009 for *Gekko*) and other statistics were consistent with the MCMC chains converging and mixing well (*SI Appendix*, Table S4).

## Discussion

To relax the assumption that all processes of biological diversification affect evolutionary lineages independently, we introduced a generalized Bayesian phylogenetic approach to inferring phylogenies with shared and multifurcating divergences. Using simulations, we found that this approach can accurately infer shared and multifurcating divergences from moderately sized datasets, while maintaining a low rate of incorrectly inferring such patterns of divergence. When we used the generalized approach to infer the evolutionary histories of two genera of gekkonid lizards across the Philippines, we found strong support against tree models assumed by current phylogenetic methods. The posterior probability of all trees with N−1 independent, bifurcating divergences was less than $2.9\times 10^{-5}$ for both genera, suggesting that trees with shared and multifurcating divergences better explain the gekkonid sequence data. It will be interesting to see if such improvement in model fit is common as the generalized tree distribution is applied to more systems, regardless of the biological processes responsible (if any).

Despite greatly expanding the number of possible topologies, we saw better MCMC behavior under the *M_G_* model (*SI Appendix*, Fig. S8), even when the *M_IB_* chains were started with the true tree and run twice as long (*SI Appendix*, Fig. S9). This could be due to the generalized tree distribution providing more ways to traverse tree space. For example, when a posterior distribution restricted to trees with independent bifurcating divergences has multiple “peaks” associated with different topologies, the generalized distribution includes tree models that are special cases of these topologies. Explicitly including these “intermediate” trees could make the posterior less rugged and allow MCMC chains to more easily traverse tree space.

By accommodating multifurcations, our generalized tree approach helped avoid the “star-tree paradox,” where arbitrary resolutions of a true polytomy can be strongly supported (Fig. 2*D* and refs. 34 and 59). Lewis et al. (34) found the same result by expanding the space of unrooted tree topologies to include multifurcations. Our results show that this solution to the star-tree paradox extends to rooted trees.

### Robustness of Coalescent Models that Assume Unlinked Characters.

Our finding that the multispecies coalescent model of Bryant et al. (40) is robust to linked characters is consistent with previous simulations using species trees with one and two tips (41, 44, 60). Our simulation results show that this robustness extends to larger trees with multifurcations and shared divergences and suggest that discarding data to avoid linked characters can have a worse effect on inference than violating the assumption of unlinked characters. This is consistent with the findings of Chifman and Kubatko (61) that quartet inference of splits in multispecies coalescent trees from SNP data were also robust to the violation of the assumption that characters are unlinked.

### Diversification of Philippine Gekkonid Lizards.

How the 7,100 islands of the Philippines accumulated one of the highest concentrations of terrestrial biodiversity on Earth (43, 62–64) has been of interest to evolutionary biologists since the founding of biogeography (65–70). Since the late Pliocene, the archipelago’s five major (and several minor) aggregate island complexes were repeatedly fragmented by interglacial rises in sea level into clusters of landmasses resembling today’s islands, followed by island fusion via land-bridge exposure as sea levels fell during glacial periods (15–19). The repeated fragmentation–fusion cycles of this insular landscape have generated a prominent hypothesis to explain the high levels of terrestrial biodiversity across the Philippines (20–30). However, there is growing evidence that 1) older tectonic processes (∼30 million to 5 million years ago [Mya]) of precursor paleoislands (29, 30, 71–73); 2) dispersal events from mainland source populations (22, 30, 68, 74); 3) repeated colonizations among islands (28, 73, 75); and 4) fine-scale in situ isolating mechanisms (28, 29, 76–79) have been important causes of diversification among and within many of the islands.

Oaks et al. (44) found support for independent divergence times among interisland pairs of *Cyrtodactylus* and *Gekko* populations from across the Philippines, suggesting that rare, over-water colonization, perhaps mediated by rafting on vegetation, might have been a more important mechanism of isolation than sea-level fragmentation in these gekkonid lizards. Our fully phylogenetic approach to this problem has allowed us to look for shared divergences across the full evolutionary history of extant populations in these clades, finding evidence for shared divergences that were missed by the pairwise approach. These results emphasize a pitfall of previous methods: Choosing pairs of populations, for comparison under previous methods for inferring shared divergences (8, 41, 80, 81), was problematic in the sense that it was somewhat arbitrary and could miss more complex patterns of shared divergences in the shared ancestry of the taxa under study.

We recognize that our use of secondary calibrations to convert the timescale of the diversification of each genus into millions of years is error-prone and should not be used to tie estimated shared divergences to specific geological or climatic events. However, given how recent most of the estimated shared divergences are for both *Cyrtodactylus* and *Gekko* (Fig. 6 and *SI Appendix*, Table S3), it is unlikely that the magnitude of error from our calibrations is great enough such that the true timing of these divergences would predate the late Pliocene. Thus, we conclude that these estimated divergences are consistent with predictions of the model of diversification based on Plio-Pleistocene interglacial fragmentation of the islands. Given that the numbers of shared or multifurcating divergence events estimated within this time frame are relatively low for both *Cyrtodactylus* and *Gekko* (six and five, respectively) and have weak to moderate support, our findings also are consistent with accumulating evidence that a number of complex processes of diversification have played important roles in shaping the distribution of life across the Philippines, not just paleo-island fragmentation.

A simultaneous analysis involving broader taxonomic sampling of Philippine gekkonids [e.g., *Gekko*, *Cyrtodactylus*, *Pseudogekko*, *Lepidodactylus*, and *Luperosaurus* (82)] would likely reveal support for an increased number of shared divergences across the archipelago, including older divergences predicted by geological processes that predate the Plio-Pleistocene interglacial fragmentations. When comparing our results between *Cyrtodactylus* and *Gekko*, we see some patterns suggestive of such shared diversification. For example, early divergences in both genera show patterns consistent with arrival into the archipelago, and subsequent diversification, via the Palawan Island Arc (29, 72). Results from both genera support a pattern where a clade, sister to species endemic to the Palawan microcontinental block, began diversifying across the oceanic islands of the Philippines ∼25 to 20 Mya (Fig. 6). Among the divergences estimated to have occurred within the last 2 My, there also appear to be regional consistencies in when and where lineages were diversifying in the Philippines, including population-level diversification for the widespread *Cyrtodactylus philippinicus* and *Gekko mindorensis* within and among the Mindoro and West Visayan faunal regions in the central Philippines (Fig. 6, *SI Appendix*, Figs. S14 and S15, and refs. 29 and 83). Regardless of temporal concordance among divergences, the results of this work further support Philippine species within both focal clades having originated in the archipelago as a result of one or more faunal exchanges between oceanic portions of the Philippines associated historically with the Philippine mobile belt and the Palawan microcontinental block (43, 84).

Currently, broader taxonomic analyses are limited by a simplifying assumption of phycoeval that mutation rates are constant across the tree. We sought to minimize the effects of violations of this assumption by analyzing the two gekkonid genera separately. The Philippine species in each genus are closely related (the posterior mean root age in expected substitutions per site for *Cyrtodactylus* and *Gekko* was 0.012 and 0.013, respectively) and share similar natural histories, so an assumption of a similar rate of mutation across the populations we sampled within each genus seems reasonable. Future developments of phycoeval allowing the rate to vary across the phylogeny would be an obvious way to improve our current implementation and make it more generally applicable to a greater diversity of systems.

### Future Directions.

Given that processes of codiversification are of interest to fields as diverse as biogeography, epidemiology, and genome evolution, we hope that the generalized tree model offers a statistical framework for studying these processes across the life sciences. To help achieve this, there are several ways to improve upon our current implementation of this approach. Allowing the generalized tree model and associated MCMC algorithms to be coupled with a diverse set of phylogenetic likelihood models is an obvious way to expand its applicability to more data types and systems. The independence of the tree model and MCMC algorithms from the likelihood function makes this relatively straightforward. Similarly, our approach can be extended to accommodate tips sampled through time (85–88) and “relaxed-clock” models (89–91). The former would allow for fossil and epidemiological data, and the latter would allow it to be applied to diverse sets of taxa that are expected to vary in their rates of mutation.

As we alluded to above when discussing MCMC behavior, expanding the set of tree models to include all possible nonreticulating topologies with 1 to N−1 divergence times could have important implications for the joint posterior distribution of phylogenetic models. We suggest that posteriors that are rugged under a tree model with strictly independent and bifurcating divergences might be smoother under a generalized tree model, but more formal theoretical work to characterize this joint space is needed.

Lastly, the distribution we used over the generalized tree space (uniform over topologies with beta-distributed node heights) is motivated by mathematical convenience, rather than inspired by biological processes. Process-based models, like a generalized birth–death model, could provide additional insights. In addition to inferring phylogenies with shared or multifurcating divergences, process-based models would allow us to infer the macroevolutionary parameters that govern the rate of such divergences.

## Materials and Methods

### Generalized Tree Model.

Let *T* represent a rooted, potentially multifurcating tree topology with *N* tips and n(t) internal nodes $t=t_{1},t_{2},\ldots t_{n(t)}$, where n(t) can range from 1 (the “comb” tree) to N−1 (fully bifurcating, independent divergences). Each internal node *T* is assigned to one divergence time τ, which it may share with other internal nodes in the tree. We will use $\tau =\tau_{1},\ldots ,\tau_{n(\tau )}$ to represent n(τ) divergence times, where n(τ) can also range from 1 to N−1, every τ has at least one node assigned to it, and every node maps to a divergence time more recent than its parent (*SI Appendix*, Fig. S17).

To formalize a distribution across this space of generalized trees, we assume that all possible topologies (*T*) are equally probable (see *SI Appendix*, Fig. S1 for an example of the sample space of topologies). We also assume that the age of the root node follows a parametric distribution (e.g., a gamma distribution), and each of the other divergence times is beta-distributed between the present ($\tau_{0}$) and the height of the youngest parent of a node mapped to the divergence time (*SI Appendix*, Fig. S17). This was inspired by and related to the Dirichlet distribution on divergence times of Kishino et al. (92), but we use beta distributions to make it easier to deal with the fact that under our generalized tree model, multiple nodes can be mapped to each divergence time. For additional flexibility, we allow a distribution to be placed on the alpha parameter of the beta distributions of all the nonroot divergence times, which we denote as $\alpha_{\tau }$.

### Likelihood Model.

To perform Bayesian phylogenetic inference under the generalized tree model, it can be coupled with any function for calculating the probability of data evolving along a tree. This means it can be coupled with any data type and associated phylogenetic likelihood function. Even if the likelihood function does not explicitly accommodate multifurcations, these can be treated as a series of arbitrary bifurcations with branches of zero length to obtain the same likelihood of the tree.

Here, we couple the generalized tree model with a multispecies coalescent model that allows the likelihood of any species tree to be estimated directly from biallelic character data, while analytically integrating out all possible gene trees and character-substitution histories along those gene trees. Below, we give a brief overview of this model; for a full description of this likelihood model, please see Bryant et al. (40), and see Oaks (41) for a correction when only variable characters are analyzed.

#### The data.

From *N* species for which we wish to infer a phylogeny, we assume that we have collected orthologous, biallelic genetic characters. By “biallelic,” we mean that each character has, at most, two states, which we refer to as “red” and “green” following Bryant et al. (40). For each character from each species, we have collected *N* copies of the locus, *r* of which are copies of the red allele. We will use n and r to denote allele counts for one character from all *N* species; i.e., $n,r=\{(n_{1},r_{1}),(n_{2},r_{2}),\ldots (n_{N},r_{N})\}$. We use D to represent these allele counts across all the characters.

#### The evolution of characters.

We assume each character evolved along a gene tree (*g*) according to a finite-characters, continuous-time Markov chain (CTMC) model, and the gene tree of each character is independent of the others, conditional on the species tree (i.e., the characters are effectively unlinked). We use *u* and *v* to denote the relative rate of mutating from the red to green state and vice versa, respectively, as a character evolves along a gene tree, forward in time (40, 41). Thus, π=u/(u+v) is the stationary frequency of the green state. We denote the overall rate of mutation as *μ*, which we assume is constant across the tree (i.e., a “strict clock”). Because evolutionary change is the product of *μ* and time, when μ=1, time is measured in units of expected substitutions per character. If a mutation rate per character per unit of time is given, then time is measured in those units (e.g., generations or years).

#### The evolution of gene trees.

We assume that the gene trees of each character branched according to a multispecies coalescent model within a single, shared, generalized species tree, where each branch *i* represents a population with a constant effective size $N_{e}^{i}$ (40, 93–96). We use $N_{e}$ to denote the effective population sizes for all branches in the generalized tree, with topology *T* and divergence times τ; $N_{e}=N_{e}^{1},N_{e}^{2},\ldots ,N_{e}^{n(t)+N}$, where n(t)+N is equal to the number of branches in the tree.

#### The likelihood.

Using the work of Bryant et al. (40), we analytically integrate over all possible gene trees and character-substitution histories to compute the likelihood of the species tree directly from all *m* biallelic characters under a multipopulation coalescent model (94, 97, 98),

[1] $$p(D\;|\;T,\tau ,N_{e},\mu ,\pi )=\prod_{i=1}^{m}p(n_{i},r_{i}\;|\;T,\tau ,N_{e},\mu ,\pi ).$$

To accommodate multifurcations, we used recursion and equation 19 of Bryant et al. (40). This equation shows how to obtain the conditional probabilities at the bottom of an ancestral branch by merging the conditional probabilities at the top of its two descendant branches. At a multifurcation, we recursively apply equation 19 of Bryant et al. (40) to merge the conditional probabilities of each descendant branch in arbitrary order. We confirmed that this recursion returns an identical likelihood as treating the multifurcation as a series of bifurcations with zero-length branches.

### Bayesian Inference.

The joint posterior probability distribution of the tree (with potential shared and multifurcating divergences) and other model parameters is given in Eq. **2**.

[2] $$\begin{array}{l} p(T,\tau ,\alpha_{\tau },N_{e},\mu ,\pi \;|\;D)\\ \;=\frac{p(D\;|\;T,\tau ,N_{e},\mu ,\pi )p(T)p(\tau \;|\;T,\alpha_{\tau })p(N_{e})p(\mu )p(\pi )p(\alpha_{\tau })}{p(D)}. \end{array}$$

#### Priors.

We use the generalized tree distribution described above as the prior on the topology (*T*) and divergence times (τ). For all of our analyses below, we 1) set the alpha parameter of the beta distributions on nonroot divergence times ($\alpha_{\tau }$) to one; 2) set the mutation rate (*μ*) to one, so that time is in units of expected substitutions per character; 3) assume that one gamma-distributed effective population size is shared across all the branches of the species tree; and 4) set the stationary frequencies of the two character states to be equal (π=0.5), making our CTMC model of character evolution a two-state equivalent to the “JC69” model of nucleotide substitution (99).

### Approximating the Posterior of Generalized Trees.

We use MCMC algorithms (37–39) to sample from the joint posterior in Eq. **2**. To sample across trees with different numbers of divergence times during the MCMC chain, we use reversible-jump MCMC (39). We also use univariate and multivariate Metropolis–Hastings algorithms (37, 38) to update the divergence times and effective population sizes. See *SI Appendix* for details and validations of our MCMC algorithms.

### Software Implementation.

We implemented the models and algorithms above for approximating the joint posterior distribution of generalized trees, divergence times, and other model parameters in the software package ecoevolity (41, 44, 60). The C++ source code for ecoevolity is freely available from GitHub (100) and includes an extensive test suite. From the C++ source code, three command-line tools are compiled for generalized tree analyses: 1) phycoeval, for performing Bayesian inference under the model described above; 2) simphycoeval, for simulating data under the model described above; and 3) sumphycoeval, for summarizing the posterior samples of generalized trees collected by phycoeval. Documentation for how to install and use the software is available at https://phyletica.org/ecoevolity/. A detailed, version-controlled history of this project, including all of the data and scripts needed to produce our results, is available as a GitHub repository (101) and was archived on Zenodo (102). We used multiple commits of ecoevolity for the analyses below, as we added features to the sumphycoeval tool (this history is documented in the project repository). However, all of our analyses can be replicated by using Version (v) 1.0.0 (Commit 2ed8d6ec) of ecoevolity.

### Simulation-Based Analyses.

#### Methods used for all our simulations (unless noted).

We used sumphycoeval to simulate datasets of 50,000 biallelic characters from one diploid individual from nine species (i.e., two copies of each character sampled from each species). Except for our simulations of linked characters described below, the characters were unlinked (i.e., each character was simulated along an independent gene tree within the species tree). For all of our simulations and analyses, we constrained the branches of the species tree to share the same mutation-scaled, diploid effective population size ($N_{e}\mu$), which we randomly drew from a gamma distribution with a shape of 20 and mean of 0.001. We used this distribution as the prior on *N_e_* in subsequent analyses of the simulated datasets. The mean of this distribution corresponds to an average number of differences per character between individuals of 0.004, which is comparable to estimates from genomic data from populations of zooplankton (103), stickleback fish (104), humans (105), and the gecko species that we analyze here (44).

We analyzed each simulated dataset under two models using phycoeval: the generalized tree model described above, which we denote as *M_G_*, and an otherwise-equivalent model that is constrained to the space of trees with independent, bifurcating divergences (i.e., trees with N−1 divergence times), which we denote as *M_IB_*. For both *M_G_* and *M_IB_*, we used a gamma-distributed prior on the age of the root node with a shape of 10 and mean of 0.2. For each dataset, we ran four independent MCMC chains for 15,000 generations, sampling every 10 generations and retaining the last 1,000 samples of each chain to approximate the posterior (4,000 total samples). For each generation, nine (equal to the number of tips) MCMC moves are randomly selected in proportion to specified weights, some of which automatically call other moves after finishing to improve mixing. Each chain started from a random bifurcating topology with no shared divergences, and the root age and other divergence times drawn randomly from their respective prior distributions.

From the 4,000 posterior samples collected for each simulated dataset, we used sumphycoeval to calculate the mean and 95% credible intervals of the root age, tree length, effective population size, and the number of divergence times and to summarize the frequency of sampled topologies, splits, nodes, and shared divergences. We define a split as a branch in the tree that “splits” the tips of the tree into two nonoverlapping subsets: those that do and do not descend from the branch. We define a node as a split with a particular set of splits that descend from it; this is necessary to summarize the frequency of multifurcations. We also used sumphycoeval to calculate the distance between every sampled tree and the true tree using the square root of the sum of squared differences in branch lengths (45, 46). To assess convergence and mixing of the chains, we used sumphycoeval to calculate the ASDSF (106) across the four chains with a minimum split frequency threshold of 10%, as well as the PSRF (the square root of equation 1.1 in ref. 51) and the ESS (52) of the log likelihood, root age, tree length, and effective population size.

#### Simulations on fixed trees.

We used simphycoeval to simulate 100 datasets on two fixed trees with nine species, one with shared and multifurcating divergences (Fig. 2*A*) and the other with only bifurcating, independent divergences (Fig. 3*A*). We analyzed each simulated dataset under models *M_G_* and *M_IB_*, both with and without constant characters; for the latter, we specified for phycoeval to correct the likelihood for only sampling variable characters (40, 41).

To explore the improved MCMC mixing under the *M_G_* model for datasets simulated on trees with shared or multifurcating divergences (*Results*), we reran the analyses under the *M_IB_* on the 100 datasets simulated on the tree shown in Fig. 2*A*. In these reanalyses under *M_IB_*, we ran the MCMC chain twice as long (30,000 generations versus 150,000) while sampling half as frequently (every 20 generations versus 10) and started each chain with the correct tree.

#### Simulations on random trees.

Using simphycoeval, we also simulated 100 datasets on trees randomly drawn from the prior distributions of the *M_G_* and *M_IB_* models. As above, we analyzed each simulated dataset with and without constant characters under the *M_G_* and *M_IB_* models. We used MCMC to sample trees randomly from the prior distributions of both models. More specifically, we used simphycoeval to 1) randomly assemble a strictly bifurcating tree with no shared divergences times; 2) run an MCMC chain of topology-changing moves for a specified number of generations (we used 1,000); and 3) draw the root age, other divergence times, and the effective population sizes randomly from their respective prior distributions. For each MCMC generation, nine (equal to the number of tips) topology-changing moves were randomly selected in proportion to specified weights.

Due to the nested beta (uniform) distributions on nonroot divergence times, some trees sampled from *M_G_* and *M_IB_* will have all or most of the divergence times close to zero. This happens when one of the oldest nonroot divergences is randomly assigned a time near zero. For example, the trees shown in *SI Appendix*, Fig. S16 *A*–*C* all have eight independent, bifurcating divergences. Given such trees, it is nearly impossible to differentiate independent divergences with a finite dataset. It is also not clear what an investigator would want phycoeval to infer, given a true tree like *SI Appendix*, Fig. S16*A*; whereas eight independent divergences is technically correct in the synthetic world of nested beta distributions, it seems an unlikely biological explanation. To avoid such extreme scenarios, we rejected any trees that had divergences times closer than 0.001 substitutions per site. This resulted in 61 and 201 trees being rejected, in order to obtain 100 trees under the *M_G_* and *M_IB_* models, respectively. Despite this arbitrary filtering threshold, challenging tree shapes remained in our sample for simulations. For example, see the trees in *SI Appendix*, Fig. S16 *D*–*F*, all with eight independent, bifurcating divergences.

#### Simulations of linked characters.

The likelihood model above assumes that characters are unlinked (i.e., they evolved along gene trees that are independent of one another conditional on the species tree). To assess the effect on inference of violating this assumption, we repeated the simulations and analyses above (for both fixed and random trees), but simulated 500 loci of 100 linked characters each (i.e., for each locus, 100 characters evolved along a shared gene tree). We used simphycoeval to simulate these datasets in two ways: 1) All 50,000 characters are simulated and retained, and 2) only (at most) 1 variable character is retained for each locus. For the latter datasets, characters are unlinked, but only (at most) 500 characters, all variable, are sampled. We analyzed all of these datasets under both the *M_G_* and *M_IB_* models. For datasets with only variable characters, we corrected the likelihood for not sampling constant characters (40, 41).

### Inference of Shared Divergences in Philippine Gekkonids.

We applied our approach to two genera of geckos, *Gekko* and *Cyrtodactylus*, sampled across the Philippine Islands. We used the RADseq data of Oaks et al. (44) available on the National Center for Biotechnology Information (NCBI) Sequence Read Archive (SRA) (Bioproject PRJNA486413, SRA Study SRP158258).

#### Assembling alignments.

We used ipyrad [v0.9.43 (107)] to assemble the RADseq reads into loci for both genera. All of the scripts and ipyrad parameter files we used to assemble the data are available in our gekkonid project repository (108) archived on Zenodo (109), and the ipyrad settings are listed in *SI Appendix*, Table S5. Using pycoevolity [v0.2.9; Commit 217dbeea (41)], we converted the ipyrad alignments into nexus format and, in the process, removed sites that had more than two character states. The final alignment for *Cyrtodactylus* contained 1,702 loci and 155,887 characters from 27 individuals, after 567 characters with more than 2 states were removed. The final alignment for *Gekko* contained 1,033 loci and 94,612 characters from 26 individuals, after 201 characters with more than 2 states were removed. Both alignments had less than 1% missing characters. The assembled data matrices for *Cyrtodactylus* and *Gekko* are available in our project repository (101), and the data associated with specimens are provided in *SI Appendix*, Tables S1 and S2.

#### Phylogenetic analyses.

When analyzing the *Cyrtodactylus* and *Gekko* character matrices with phycoeval, we 1) fixed stationary state frequencies to be equal (π=0.5), 2) set the mutation rate (*μ*) to one so that divergence times are in units of expected substitutions per site, 3) used an exponentially distributed prior with a mean of 0.01 for the age of the root, 4) set $\alpha_{\tau }=1$ so that nonroot divergence times are uniformly distributed between zero and the age of the youngest parent node, and 5) assumed a single diploid effective population size (*N_e_*) shared across the branches of the tree with a gamma-distributed prior. For the gamma prior on *N_e_*, we used a shape of 2.0 and mean of 0.0005 for *Cyrtodactylus* and a shape of 4.0 and mean of 0.0002 for *Gekko*, based on estimates of Oaks et al. (44) from the same and related species.

For both genera, we ran 25 independent MCMC chains for 15,000 generations, sampling the state of the chain every 10 generations. In each generation, phycoeval attempts *N* MCMC moves (27 and 26 for *Cyrtodactylus* and *Gekko*, respectively) randomly selected in proportion to specified weights, some of which automatically call other moves after finishing to improve mixing. For 20 of the chains, we specified for phycoeval to start from the “comb” topology (n(τ)=1). For the remaining five chains, we had phycoeval start with a random bifurcating topology with no shared divergences (n(τ)=N−1).

We used sumphycoeval to summarize the sampled values of all parameters and the frequency of sampled topologies, splits, nodes, and shared divergences. To assess convergence and mixing, we used sumphycoeval to calculate the ASDSF (106) and the PSRF (the square root of equation 1.1 in ref. 51) and ESS (52) of all parameters across all 25 MCMC chains. We present these convergence statistics in *SI Appendix*, Table S4.

To plot the trees, we used sumphycoeval to scale the branch lengths of all the sampled trees so that the posterior mean root age was 23.07 My for *Cyrtodactylus* (110) and 33.76 My for *Gekko* (111). Our goal in scaling the branch lengths to millions of years is not to test whether shared divergence events correspond with the onset of specific interglacial periods, but rather to see if shared divergences fall within the general time frame predicted by a model of sea-level-driven diversification (i.e., within the last ≈4 My).

## Supplementary Material

Supplementary File

## Data Availability

We recorded a detailed history of all our analyses for this project in a version-controlled repository, which is publicly available at GitHub [https://github.com/phyletica/phycoeval-experiments (101)]) and archived on Zenodo [https://doi.org/10.5281/zenodo.5162056 (102)]. The C++ source code for ecoevolity is freely available from GitHub [https://github.com/phyletica/ecoevolity (100)], with detailed documentation and tutorials available at https://phyletica.org/ecoevolity. All of the scripts and parameter files we used to assemble the gecko datasets are available in our gekkonid project repository [https://github.com/phyletica/gekgo (108)] that is archived on Zenodo [https://doi.org/10.5281/zenodo.5162085 (109)]. The gecko sequence reads are available on the NCBI SRA [Bioproject PRJNA486413 (112), SRA Study SRP158258 (113)].
