# Supplementary material for: Generalizing Bayesian phylogenetics to infer shared evolutionary events
Source: Proc Natl Acad Sci U S A. 2022 Jul 15;119(29):e2121036119. doi: 10.1073/pnas.2121036119 (PMC9304017; doi:10.1073/pnas.2121036119)
Supplement: Supplementary File [file pnas.2121036119.sapp.pdf]

Supporting Information

Title: Generalizing Bayesian phylogenetics to infer shared evolutionary events

Authors: Jamie R. Oaks Corresponding author: joaks@auburn.edu[1], Perry L. Wood, Jr.[1], Cameron D. Siler[2], and Rafe M. Brown[3]

[1]Department of Biological Sciences & Museum of Natural History, Auburn University, Auburn, Alabama 36849, USA
[2]Sam Noble Oklahoma Museum of Natural History and Department of Biology, University of Oklahoma, Norman, Oklahoma 73072-7029, USA
[3]Biodiversity Institute and Department of Ecology and Evolutionary Biology, University of Kansas, Lawrence, Kansas 66045, USA

Table of Contents

1 Figures referenced in main text **3**

2 Tables **19**

3 The generalized tree model **23**

4 Approximating the posterior of the generalized tree model **24**

4.1 Split-time move . . . 25
4.1.1 Drawing the new divergence time . . . 25
4.1.2 Prior ratio . . . 26
4.1.3 Hastings ratio . . . 26
4.2 Merge-times move . . . 28
4.2.1 Prior ratio . . . 28
4.2.2 Hastings ratio . . . 28
4.3 Expanding Ξ . . . 29
4.3.1 The case of all bifurcating nodes mapped to τ_i . . . 29
4.3.2 The case of a single polytomy mapped to τ_i . . . 29
4.3.3 The case when multiple nodes, including at least one polytomy, are mapped to τ_i . . . 30
4.4 Validation of Split-time and Merge-times moves . . . 32
4.5 Nested-neighbor-node-swap move . . . 33
4.5.1 Validation of move . . . 34
4.6 Divergence time slide bump move . . . 34

4.6.1 An extension to this move 38
4.6.2 Validation of this move 38

1 Figures referenced in main text

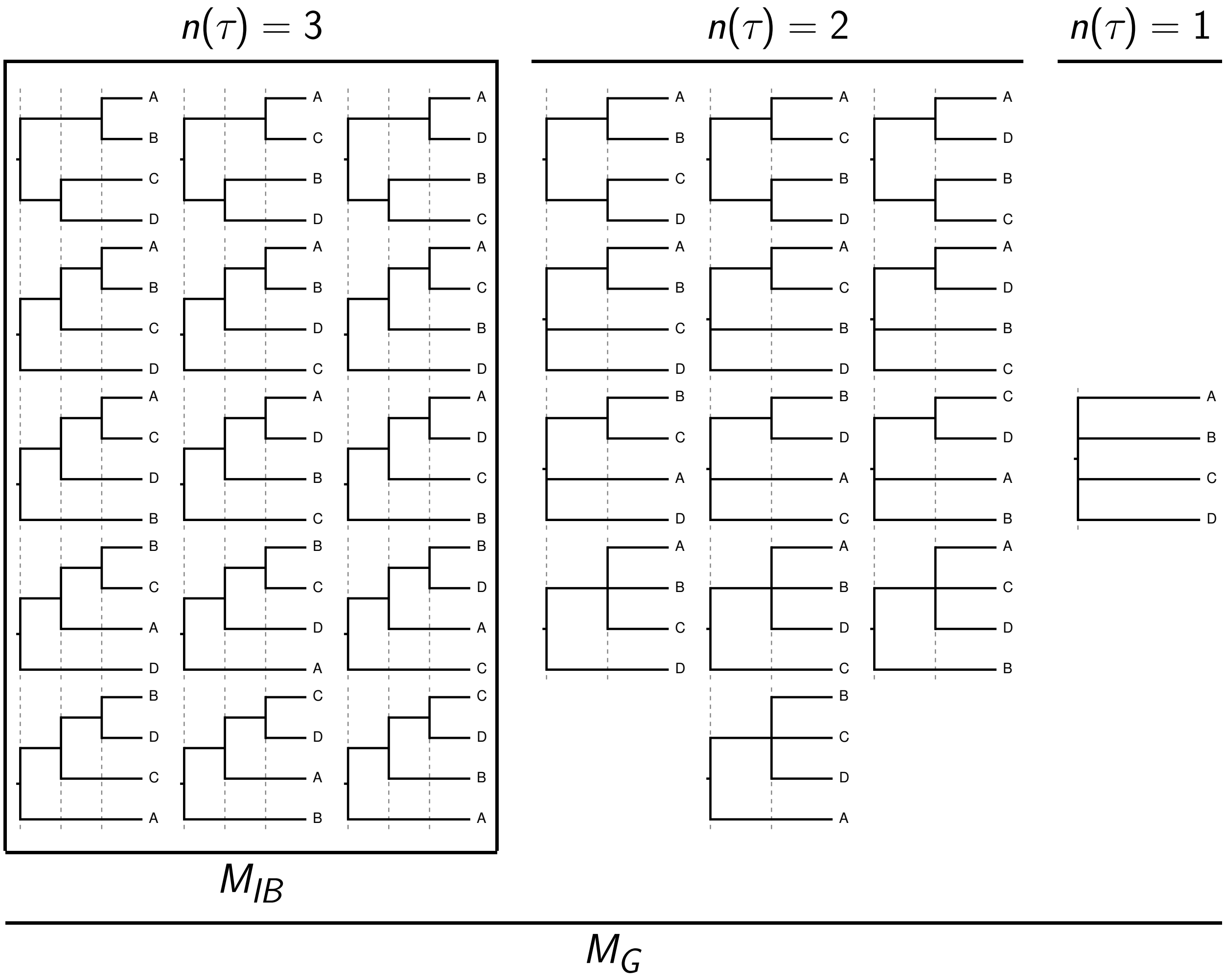

Figure S1. An example of the expanded sample space of topologies for the generalized tree distribution (M_G) versus a tree model that assumes independent, bifurcating divergences (M_{IB}). With four tips, there are 15 topologies for a M_{IB} model, all with three divergence times (within box). The M_G model has 14 additional topologies with fewer than three divergence times. Notice the internal nodes are not labeled; i.e., the rank, or order, of non-nested internal nodes do not matter. *E.g.*, in the topology at the top-left, regardless of whether A & B or C & D diverge first, it is the same topology. However, if A & B and C & D diverge at the same time, this is a different topology (or tree model) with one fewer divergence-time parameter (the topology in the upper-left of the $n(\tau) = 2$ section). Trees plotted using Gram (Version 4.0.0; Foster, 2018) and the P4 phylogenetic toolkit (Version 1.4 5742542; Foster, 2004).

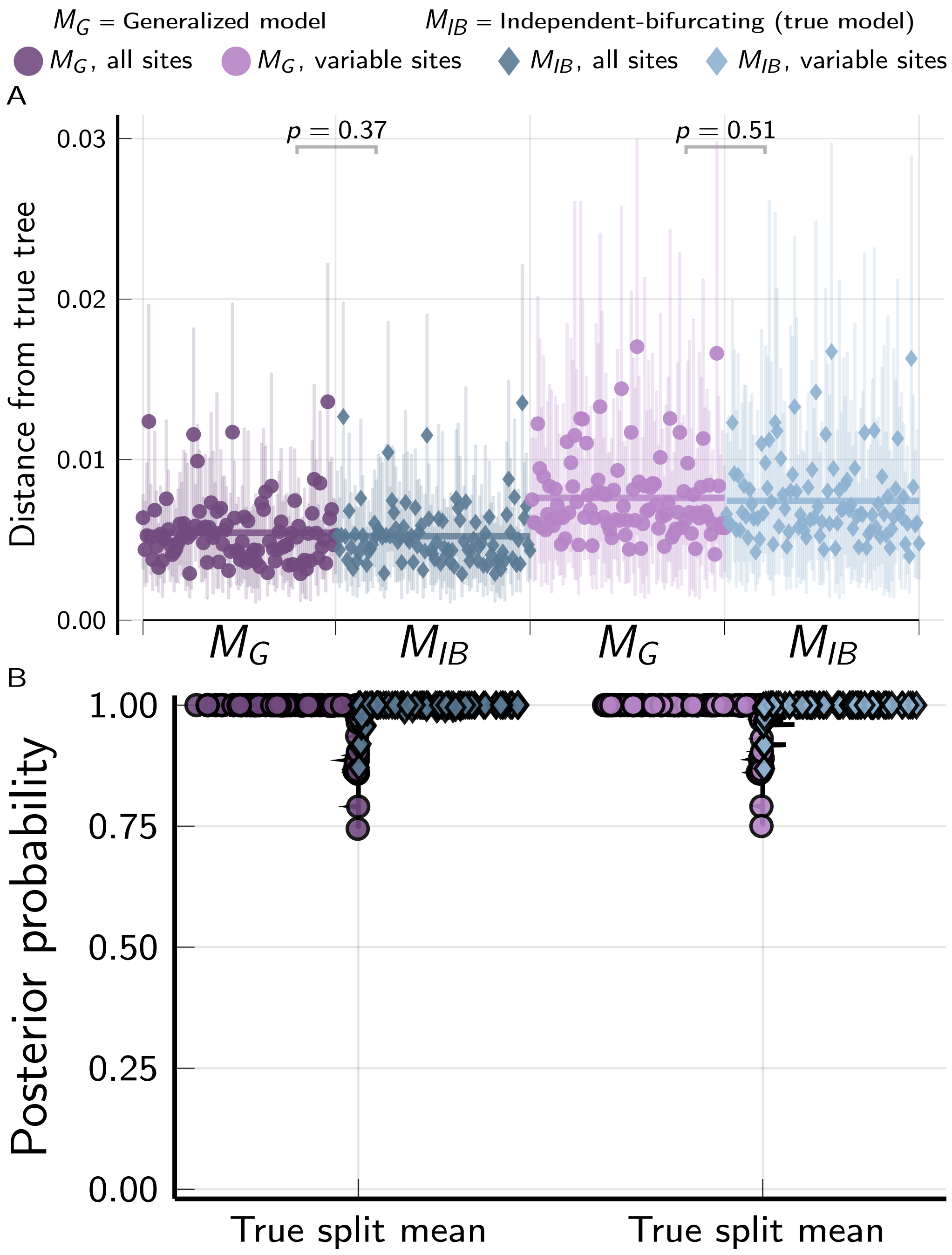

Figure S2. The performance of the M_G and M_{IB} tree models when applied to 100 data sets, each with 50,000 biallelic characters simulated on species trees randomly drawn from the M_{IB} tree distribution. (A) The square root of the sum of squared differences in branch lengths between the true tree and each posterior tree sample (Kuhner and Felsenstein, 1994); the point and bars represent the posterior mean and equal-tailed 95% credible interval, respectively. P-values are shown for Wilcoxon signed-rank tests (Wilcoxon, 1945) comparing the paired differences in tree distances between methods. (B) Violin plots comparing the mean posterior probabilities of true splits for each of the 100 simulated trees. For each simulation, the mutation-scaled effective population size ($N_e\mu$) was drawn from a gamma distribution (shape = 20, mean = 0.001) and shared across all the branches of the tree; this distribution was used as the prior in analyses. Plots created using the PGFPlotsX (Version 1.2.10, Commit 1adde3d0; Carlsson and Papp, 2021) backend of the Plots (Version 1.5.7, Commit f80ce6a2; Breloff, 2021) package in Julia (Version 1.5.4; Bezanson et al., 2017).

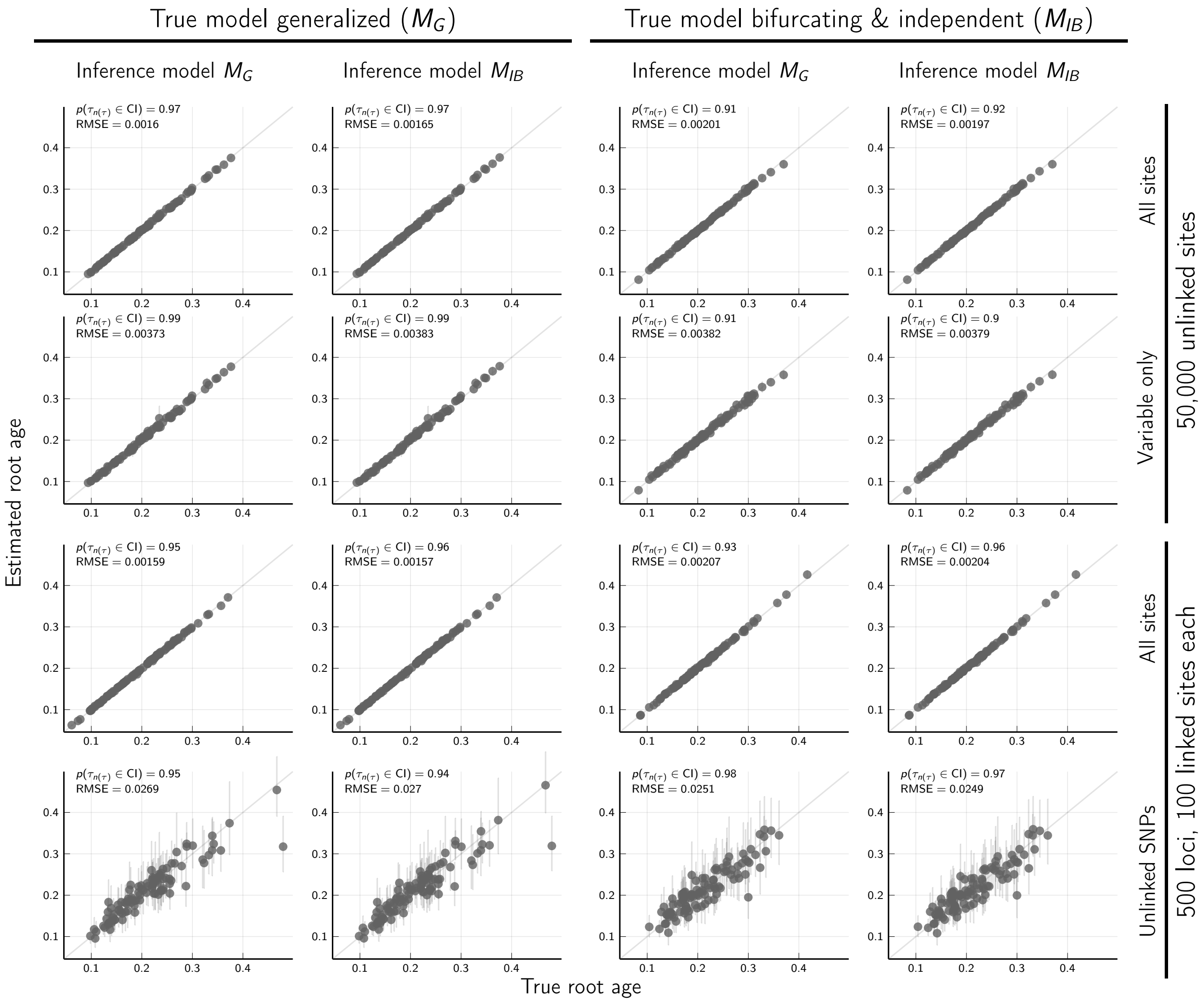

Figure S3. The accuracy and precision of the M_G and M_{IB} models at estimating the age of the root (in expected subsitutions per site) from data sets with 50,000 biallelic characters simulated on species trees randomly drawn from the M_G and M_{IB} tree distributions. Each plotted circle and associated error bars represent the posterior mean and 95% credible interval. Estimates for which the potential-scale reduction factor was greater than 1.2 (Brooks and Gelman, 1998) or the effective sample size was less than 200 are highlighted in red. Plots created using the PGFPlotsX (Version 1.2.10, Commit 1adde3d0; Carlsson and Papp, 2021) backend of the Plots (Version 1.5.7, Commit f80ce6a2; Breloff, 2021) package in Julia (Version 1.5.4; Bezanson et al., 2017).

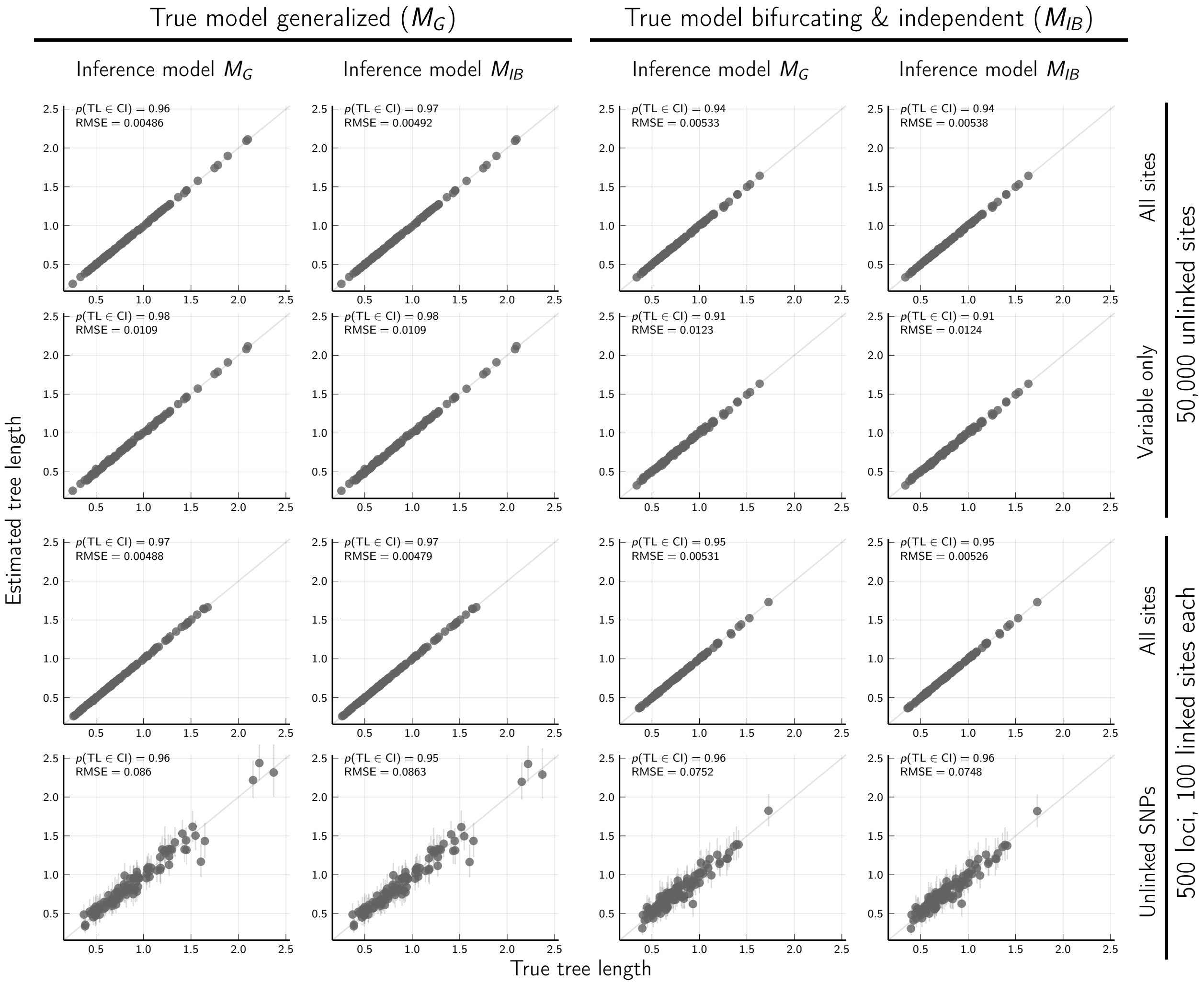

Figure S4. The accuracy and precision of the M_G and M_{IB} models at estimating the tree length (the sum of all branch lengths in units of expected substitutions per site) from data sets with 50,000 biallelic characters simulated on species trees randomly drawn from the M_G and M_{IB} tree distributions. Each plotted circle and associated error bars represent the posterior mean and 95% credible interval. Estimates for which the potential-scale reduction factor was greater than 1.2 (Brooks and Gelman, 1998) or the effective sample size was less than 200 are highlighted in red. Plots created using the PGFPlotsX (Version 1.2.10, Commit 1adde3d0; Carlsson and Papp, 2021) backend of the Plots (Version 1.5.7, Commit f80ce6a2; Breloff, 2021) package in Julia (Version 1.5.4; Bezanson et al., 2017).

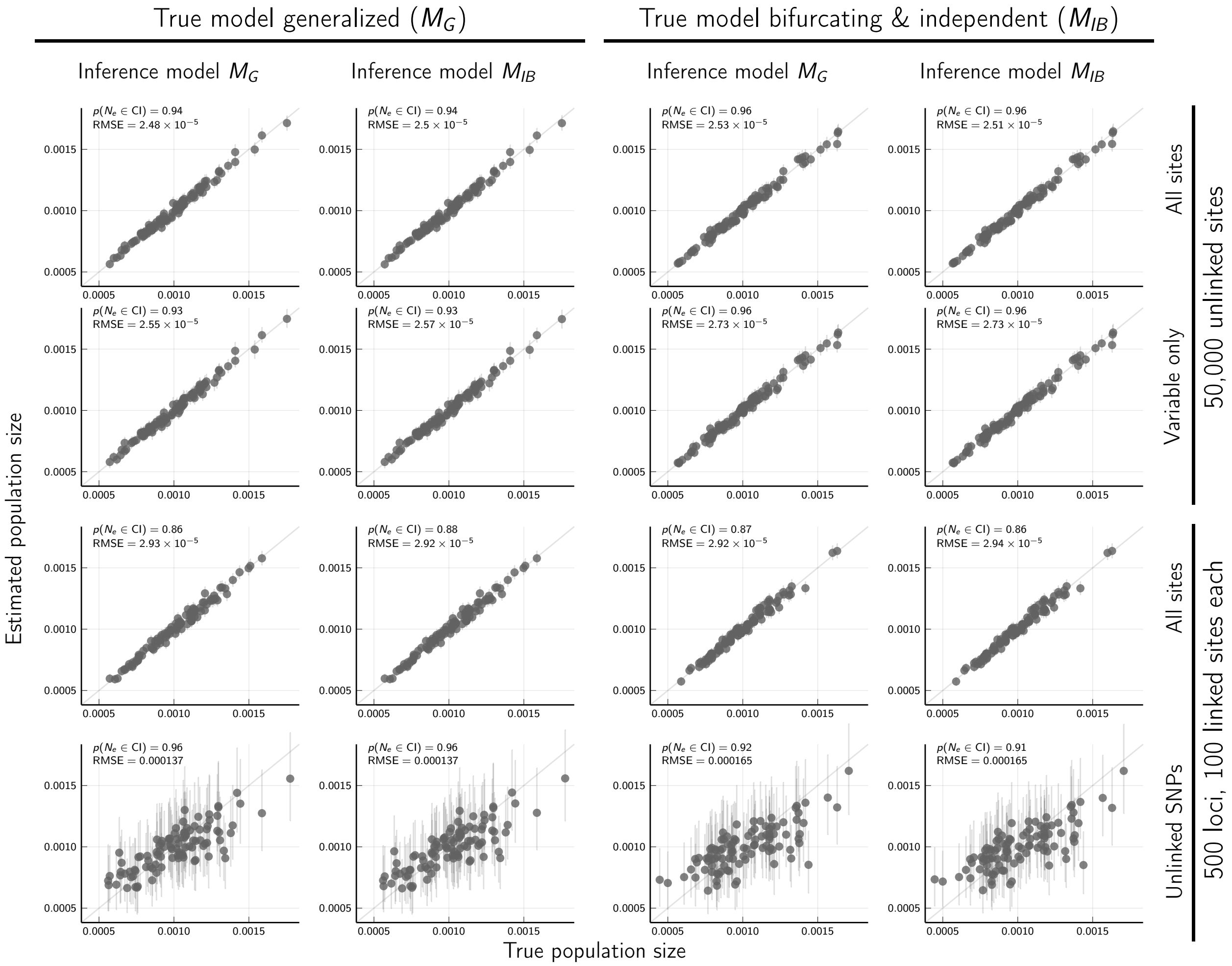

Figure S5. The accuracy and precision of the M_G and M_{IB} models at estimating the effective population size ($N_e\mu$) across the tree from data sets with 50,000 biallelic characters simulated on species trees randomly drawn from the M_G and M_{IB} tree distributions. Each plotted circle and associated error bars represent the posterior mean and 95% credible interval. Estimates for which the potential-scale reduction factor was greater than 1.2 (Brooks and Gelman, 1998) or the effective sample size was less than 200 are highlighted in red. Plots created using the PGFPlotsX (Version 1.2.10, Commit 1adde3d0; Carlsson and Papp, 2021) backend of the Plots (Version 1.5.7, Commit f80ce6a2; Breloff, 2021) package in Julia (Version 1.5.4; Bezanson et al., 2017).

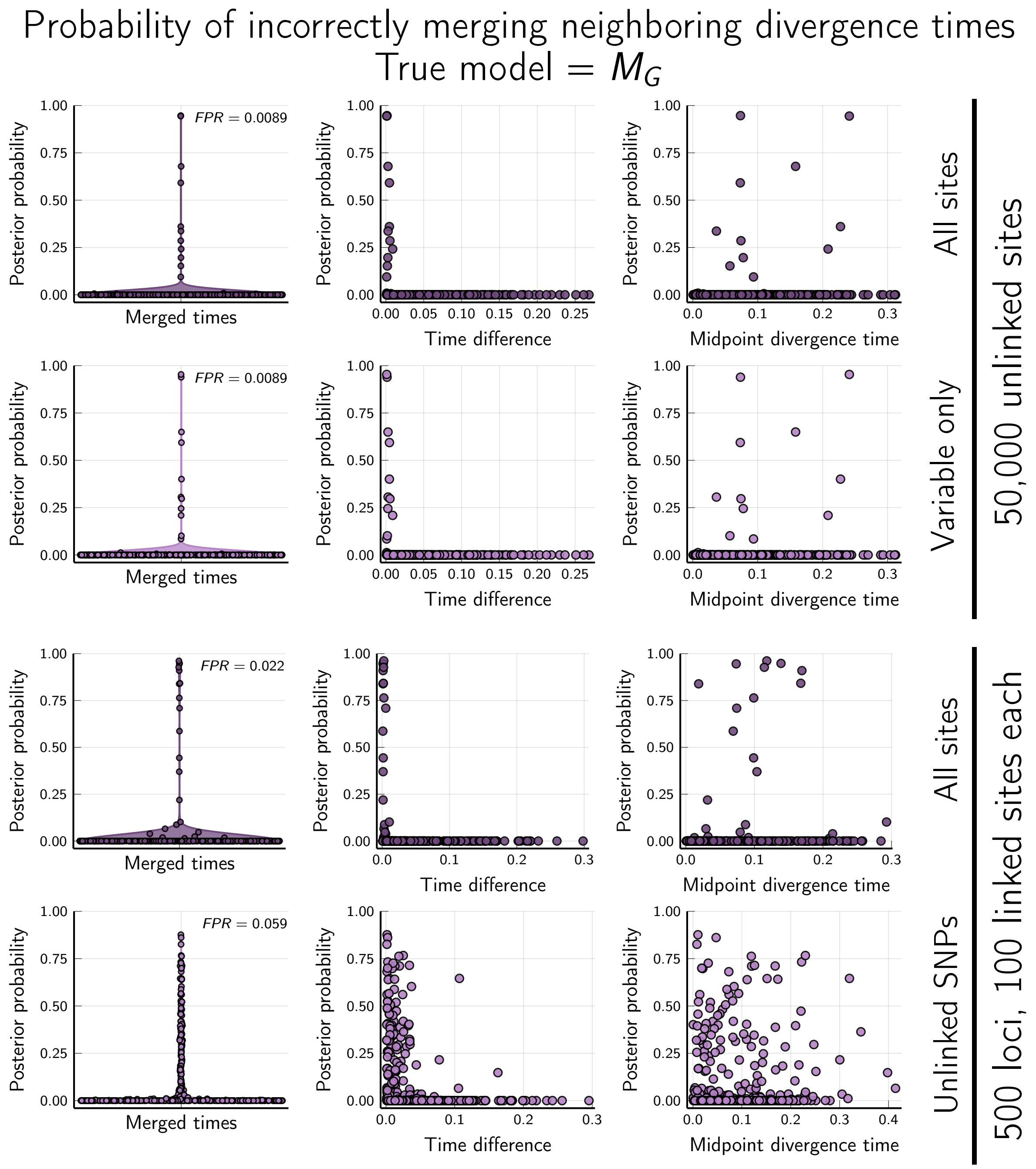

Figure S6. The M_G model has a low false positive rate (FPR; the proportion of incorrectly merged divergence times with a posterior probability > 0.5) when applied to data simulated on trees drawn from M_G with all (Row 1) or only variable (Row 2) unlinked characters, and all characters from linked loci (Row 3). Support for incorrectly merged divergence times is high only when the difference between the times is small (Column 2), and is not correlated with the age of the merged nodes (right). (Column 3; $P = 0.109$, 0.106, 0,053, and 0.068, from top to bottom for a t-test that Pearson's correlation coefficient $= 0$ using all points with posterior probability > 0). When data sets with linked loci are reduced to only one variable site per locus (Row 4), the FPR increases (left) and precision decreases (right). The top row is the same as Figure 5A–C. Time units are expected substitutions per site. Plots created using the PGFPlotsX (Version 1.2.10, Commit 1adde3d0; Carlsson and Papp, 2021) backend of the Plots (Version 1.5.7, Commit f80ce6a2; Breloff, 2021) package in Julia (Version 1.5.4; Bezanson et al., 2017).

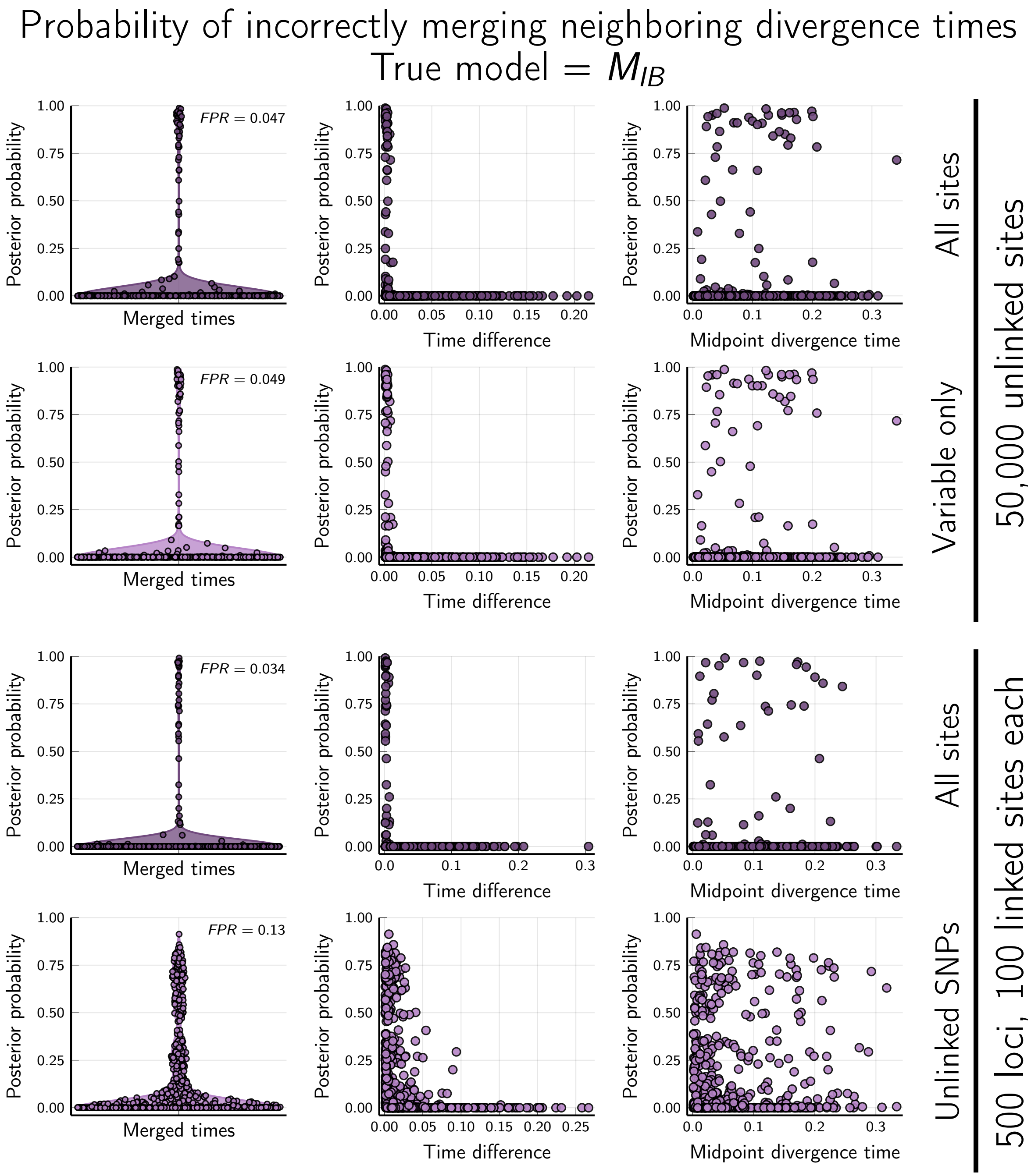

Figure S7. The M_G model has a low false positive rate (FPR; the proportion of incorrectly merged divergence times with a posterior probability $>$ 0.5) when applied to data simulated on trees drawn from M_{IB} (no shared or multifurcating divergences) with all (Row 1) or only variable (Row 2) unlinked characters, and all characters from linked loci (Row 3). Support for incorrectly merged divergence times is high only when the difference between the times is small (Column 2), and is not correlated with the age of the merged nodes (right). (Column 3; $P = 0.25$, 0.29, 0,11, and 0.11, from top to bottom for a t-test that Pearson's correlation coefficient $= 0$ using all points with posterior probability > 0). When data sets with linked loci are reduced to only one variable site per locus (Row 4), the FPR increases (left) and precision decreases (right). The top row is the same as Figure 5D–F. Time units are expected substitutions per site. Plots created using the PGFPlotsX (Version 1.2.10, Commit 1adde3d0; Carlsson and Papp, 2021) backend of the Plots (Version 1.5.7, Commit f80ce6a2; Breloff, 2021) package in Julia (Version 1.5.4; Bezanson et al., 2017).

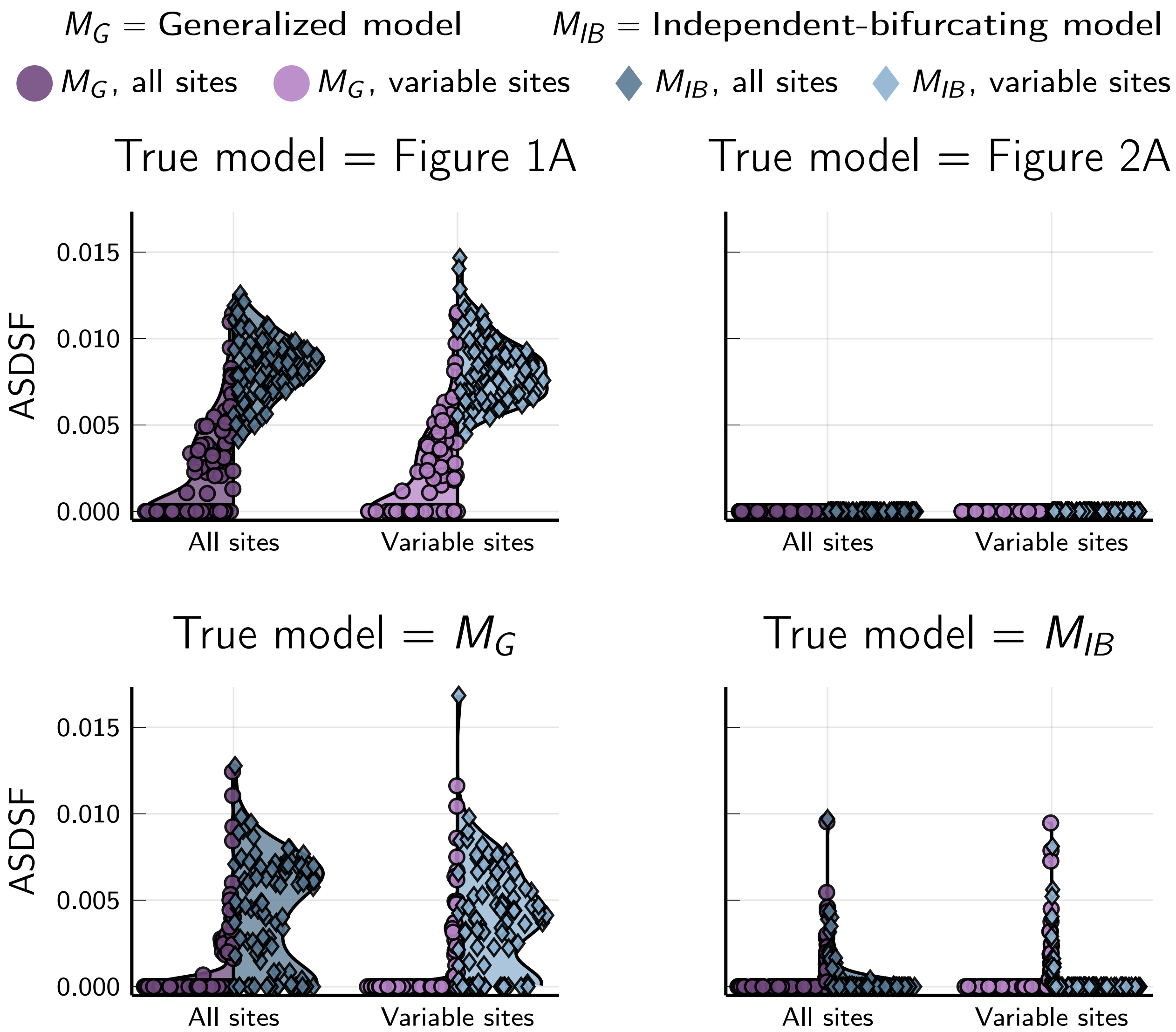

Figure S8. Markov chain Monte Carlo (MCMC) sampling yielded better convergence and mixing among chains under the generalized tree model when there were shared or multifurcating divergences. Convergence and mixing of sampled trees is summarized using the average standard deviation of split frequencies (ASDSF; Lakner et al., 2008) across the four chains of each analysis; smaller standard deviations across chains indicate better sampling behavior. Plots created using the PGFPlotsX (Version 1.2.10, Commit 1adde3d0; Carlsson and Papp, 2021) backend of the Plots (Version 1.5.7, Commit f80ce6a2; Breloff, 2021) package in Julia (Version 1.5.4; Bezanson et al., 2017).

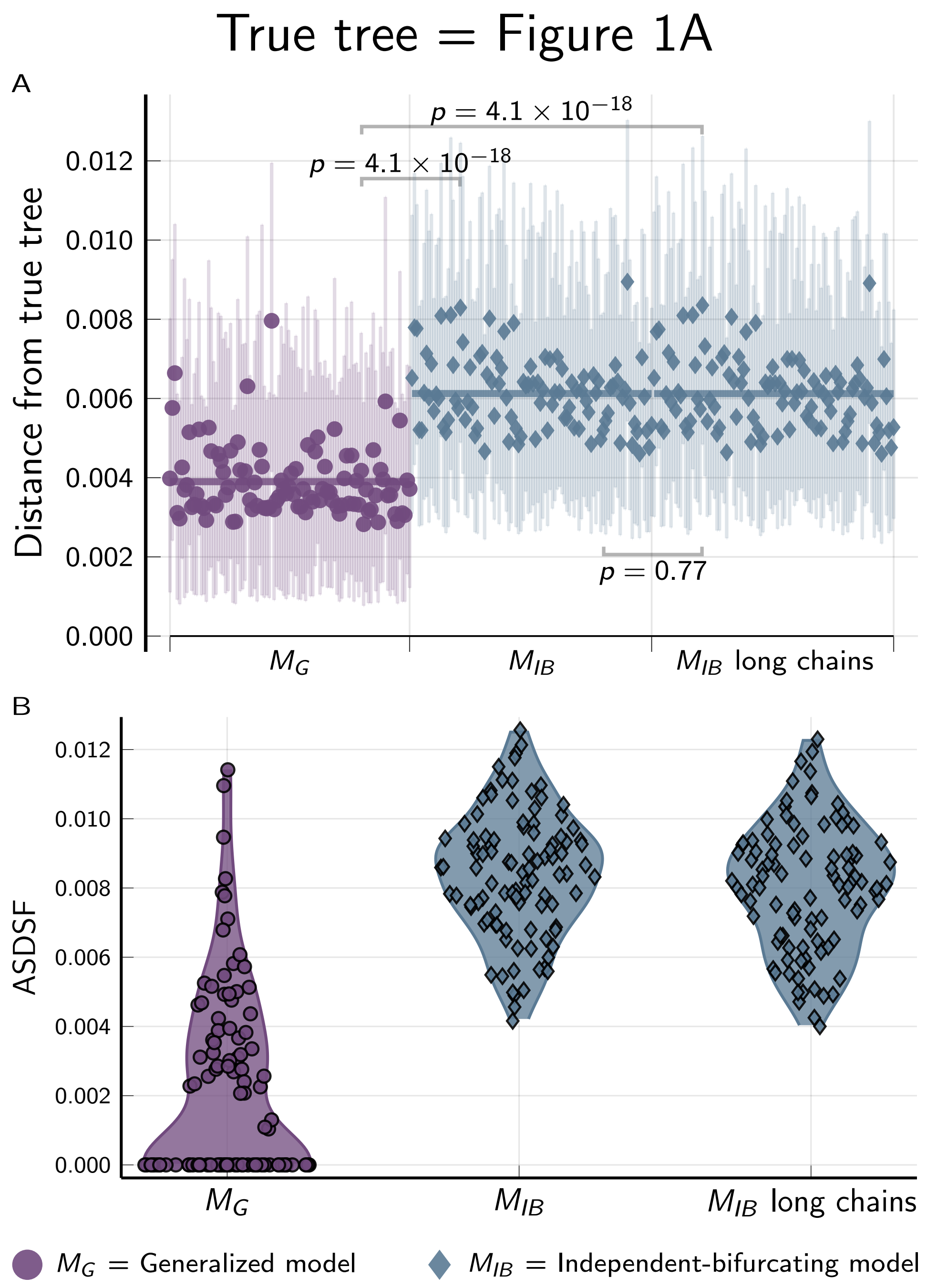

Figure S9. The (A) accuracy and (B) Markov chain Monte Carlo (MCMC) sampling efficiency of the M_G tree model is better than M_{IB}, even when the MCMC chains for M_{IB} are started with the correct tree and are run twice as long and sampled half as frequently ("long chains"; 30,000 generations, sampling every 20). Results from 100 data sets simulated on the species tree shown in Figure 2A. (A) The square root of the sum of squared differences in branch lengths between the true tree and each posterior tree sample (Kuhner and Felsenstein, 1994); the point and bars represent the posterior mean and equal-tailed 95% credible interval, respectively. P-values are shown for Wilcoxon signed-rank tests (Wilcoxon, 1945) comparing the paired differences in tree distances between methods. (B) Violin plots of the average standard deviation of split frequencies (ASDSF; Lakner et al., 2008, ; smaller is better) across the four chains of each analysis. The M_G and M_{IB} results (left and center) are the same shown in Figure 2B and Figure S8. Plots created using the PGFPlotsX (Version 1.2.10, Commit 1adde3d0; Carlsson and Papp, 2021) backend of the Plots (Version 1.5.7, Commit f80ce6a2; Breloff, 2021) package in Julia (Version 1.5.4; Bezanson et al., 2017).

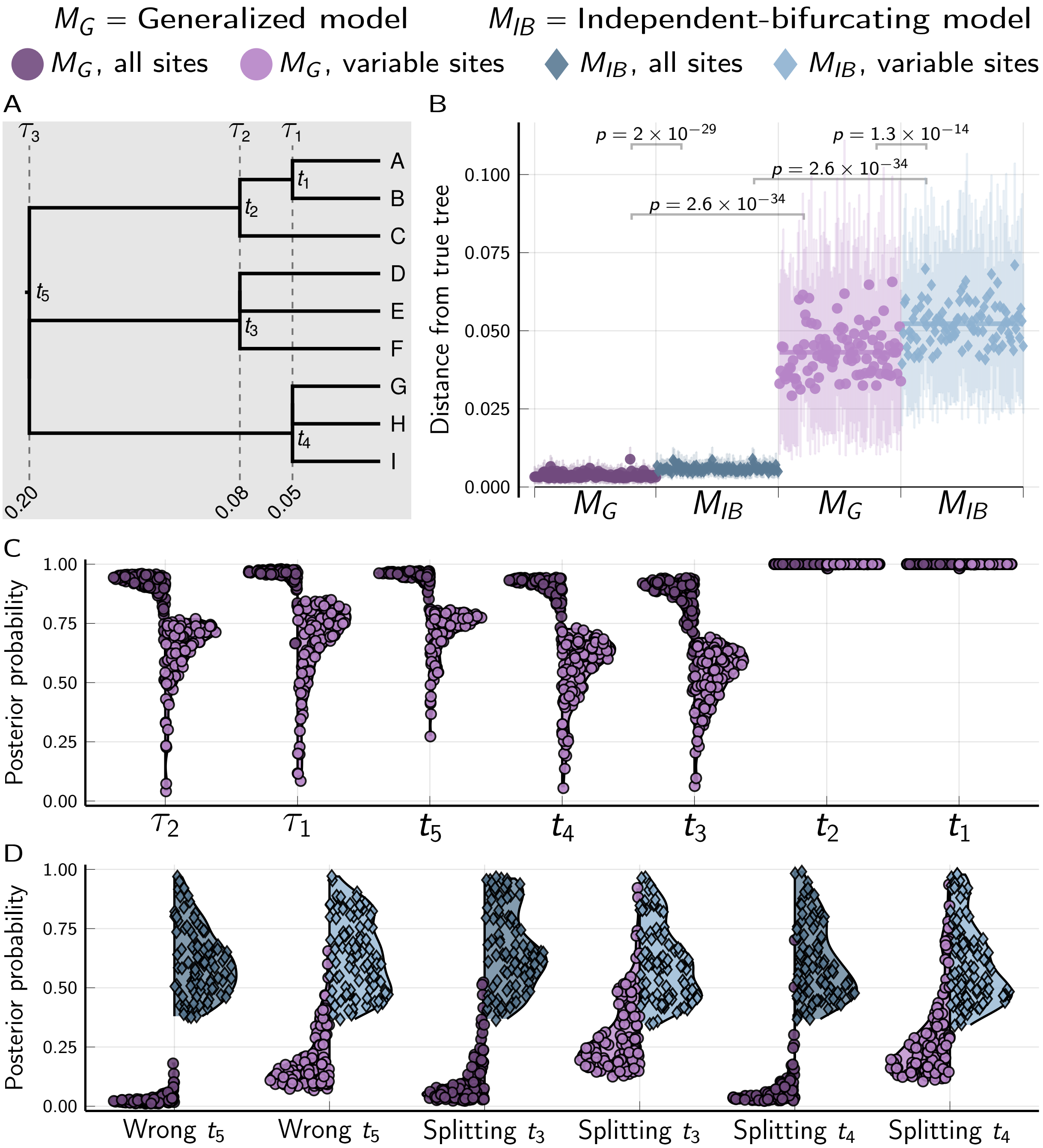

Figure S10. Results of analyses of 100 data sets with linked loci simulated along the species tree shown in (A). Each simulated data set comprised 500 loci with 100 biallelic characters. P-values are shown for Mann-Whitney U tests Mann and Whitney (1947) comparing the differences in tree distances between methods. For each simulation, the mutation-scaled effective population size ($N_e\mu$) was drawn from a gamma distribution (shape = 20, mean = 0.001) and shared across all the branches of the tree; this distribution was used as the prior in analyses. Tree plotted using Gram (Version 4.0.0, Commit 02286362; Foster, 2018) and the P4 phylogenetic toolkit (Version 1.4, Commit d9c8d1b1; Foster, 2004). Other plots created using the PGFPlotsX (Version 1.2.10, Commit 1adde3d0; Carlsson and Papp, 2021) backend of the Plots (Version 1.5.7, Commit f80ce6a2; Breloff, 2021) package in Julia (Version 1.5.4; Bezanson et al., 2017).

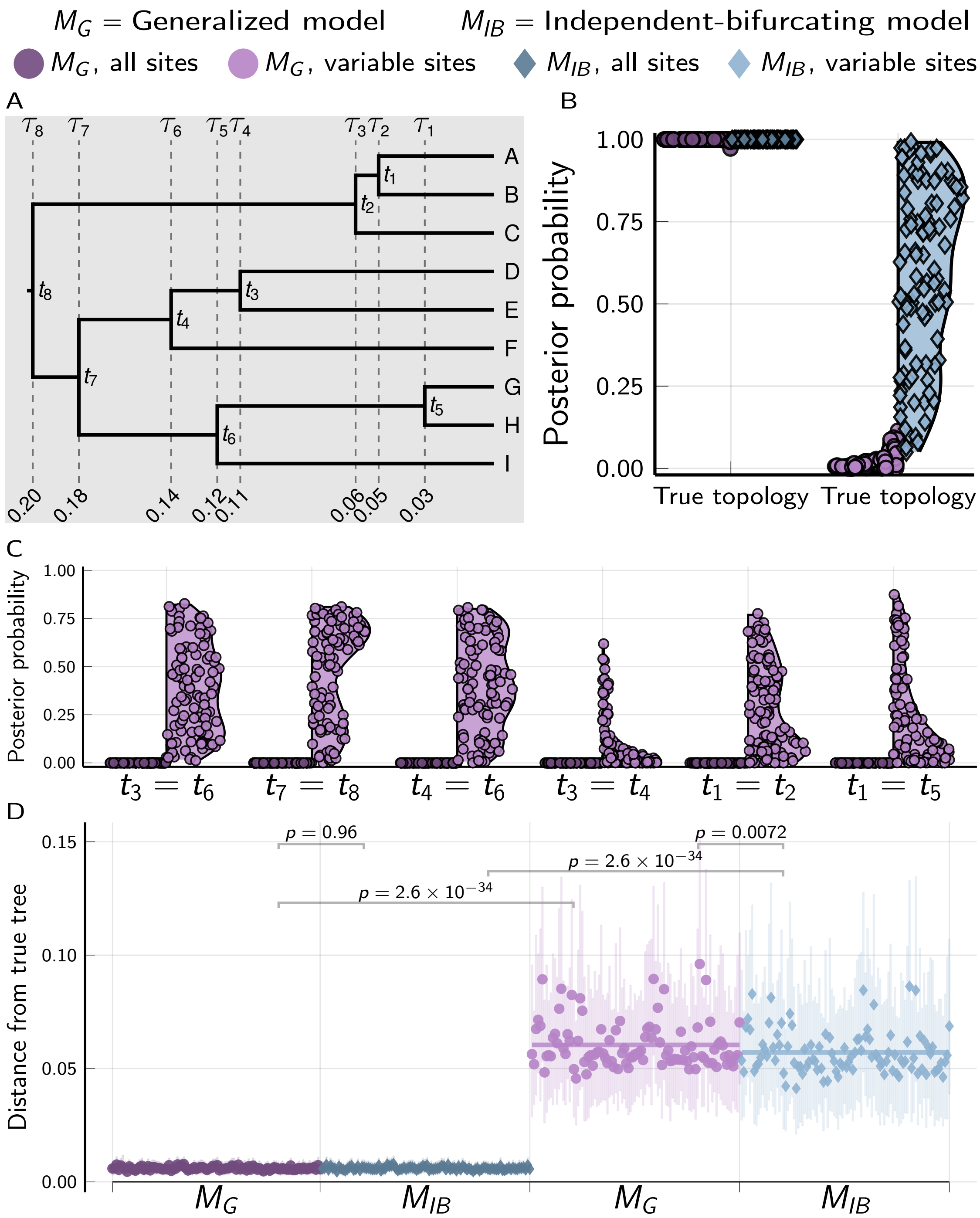

Figure S11. Results of analyses of 100 data sets with linked loci simulated along the species tree shown in (A). Each simulated data set comprised 500 loci with 100 biallelic characters. P-values are shown for Mann-Whitney U tests (Mann and Whitney, 1947) comparing the differences in tree distances between methods. For each simulation, the mutation-scaled effective population size ($N_e\mu$) was drawn from a gamma distribution (shape = 20, mean = 0.001) and shared across all the branches of the tree; this distribution was used as the prior in analyses. Tree plotted using Gram (Version 4.0.0, Commit 02286362; Foster, 2018) and the P4 phylogenetic toolkit (Version 1.4, Commit d9c8d1b1; Foster, 2004). Other plots created using the PGFPlotsX (Version 1.2.10, Commit 1adde3d0; Carlsson and Papp, 2021) backend of the Plots (Version 1.5.7, Commit f80ce6a2; Breloff, 2021) package in Julia (Version 1.5.4; Bezanson et al., 2017).

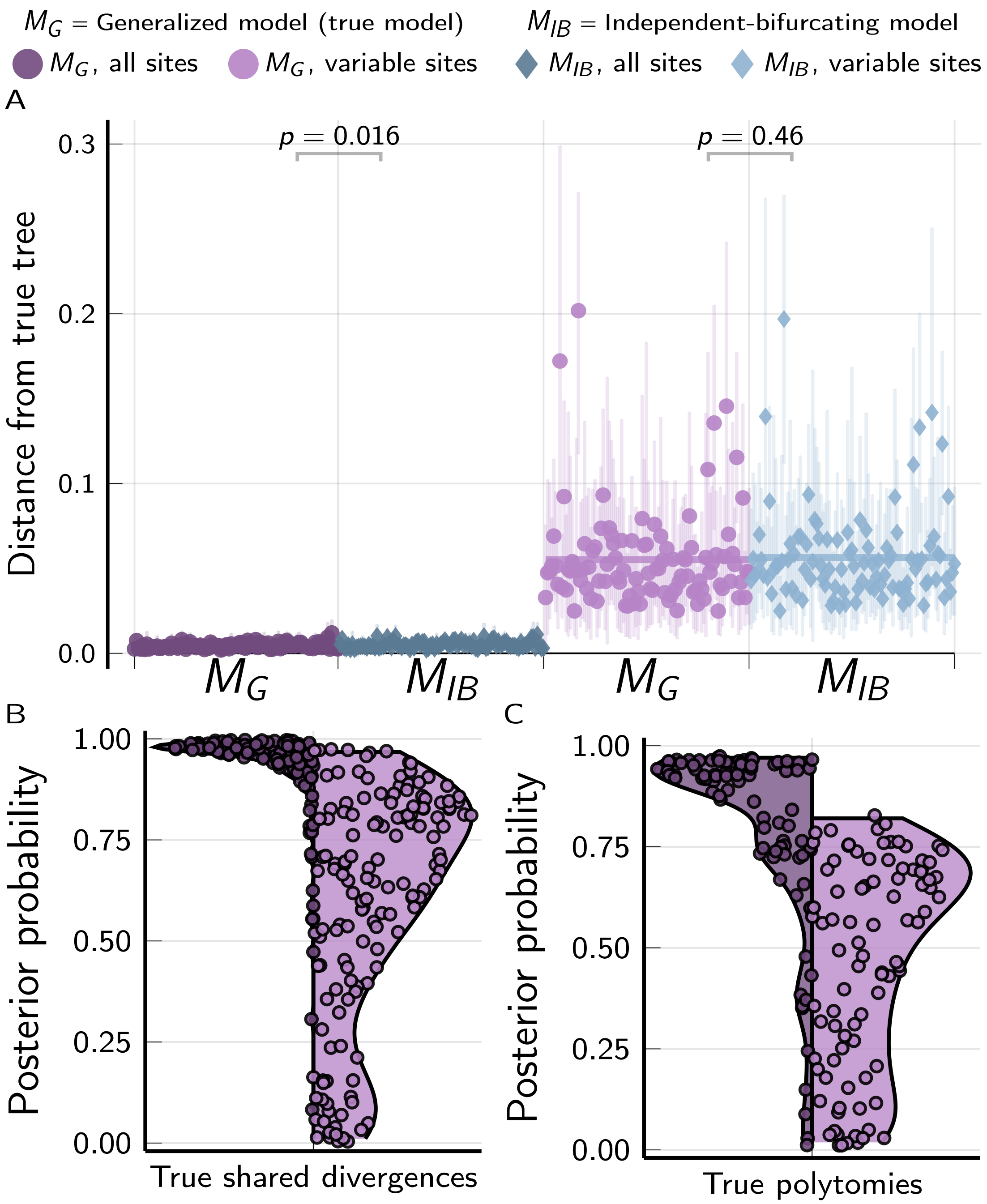

Figure S12. The performance of the M_G and M_{IB} tree models when applied to 100 data sets with 500 loci (each with 100 linked characters) simulated on species trees randomly drawn from the M_G tree distribution. For each simulation, the mutation-scaled effective population size ($N_e\mu$) was drawn from a gamma distribution (shape = 20, mean = 0.001) and shared across all the branches of the tree; this distribution was used as the prior in analyses. Plots created using the PGFPlotsX (Version 1.2.10, Commit 1adde3d0; Carlsson and Papp, 2021) backend of the Plots (Version 1.5.7, Commit f80ce6a2; Breloff, 2021) package in Julia (Version 1.5.4; Bezanson et al., 2017).

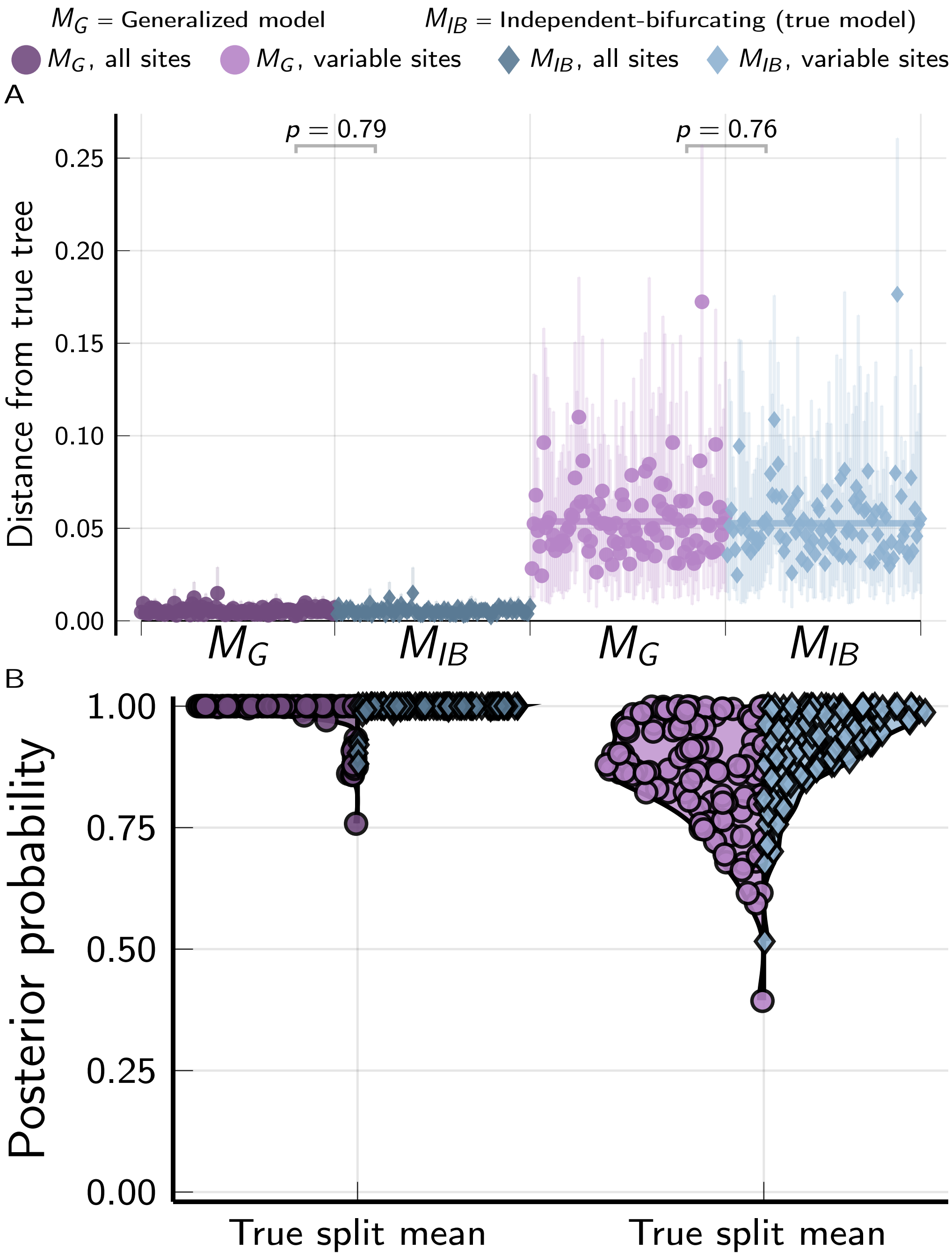

Figure S13. The performance of the M_G and M_{IB} tree models when applied to 100 data sets with 500 loci (each with 100 linked characters) simulated on species trees randomly drawn from the M_{IB} tree distribution. For each simulation, the mutation-scaled effective population size ($N_e\mu$) was drawn from a gamma distribution (shape = 20, mean = 0.001) and shared across all the branches of the tree; this distribution was used as the prior in analyses. Plots created using the PGFPlotsX (Version 1.2.10, Commit 1adde3d0; Carlsson and Papp, 2021) backend of the Plots (Version 1.5.7, Commit f80ce6a2; Breloff, 2021) package in Julia (Version 1.5.4; Bezanson et al., 2017).

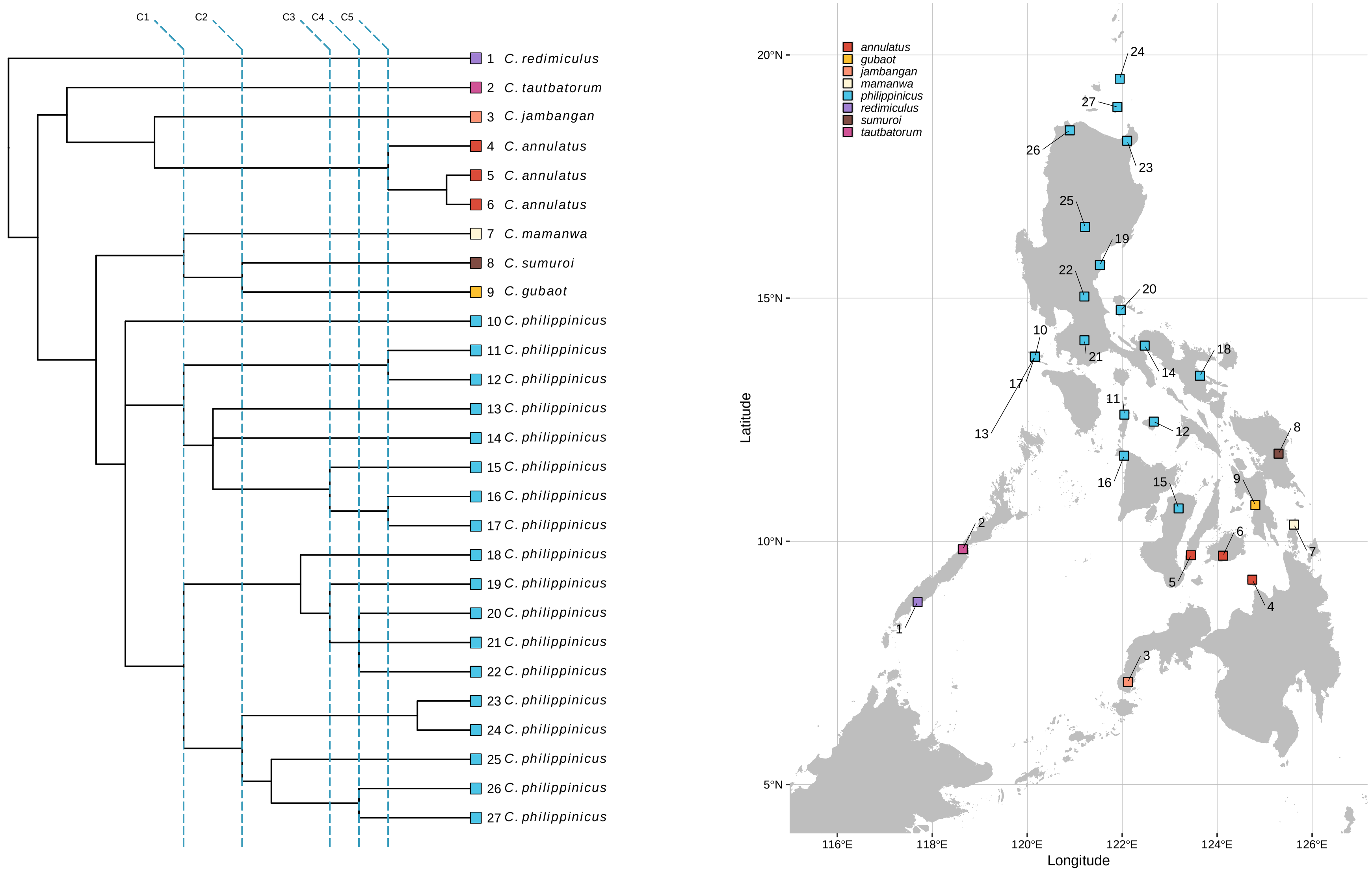

Figure S14. The maximum *a posteriori* (MAP) topology from Figure 6A for *Cyrtodactylus* shown without branch lengths to make it clearer which clades are involved. Shared divergences indicated by dashed lines, with labels shown along the top that correspond to rows in Table S3, where the divergences are summarized. Created using ggplot2 (v3.3.5; Wickham, 2016), ggtree (v3.1.0; Yu et al., 2017), treeio (v1.17.0; Wang et al., 2019), cowplot (v1.1.1; Wilke, 2020), and ggrepel (v0.9.1; Slowikowski, 2020). Link to nexus-formatted annotated MAP tree: *Cyrtodactylus*.

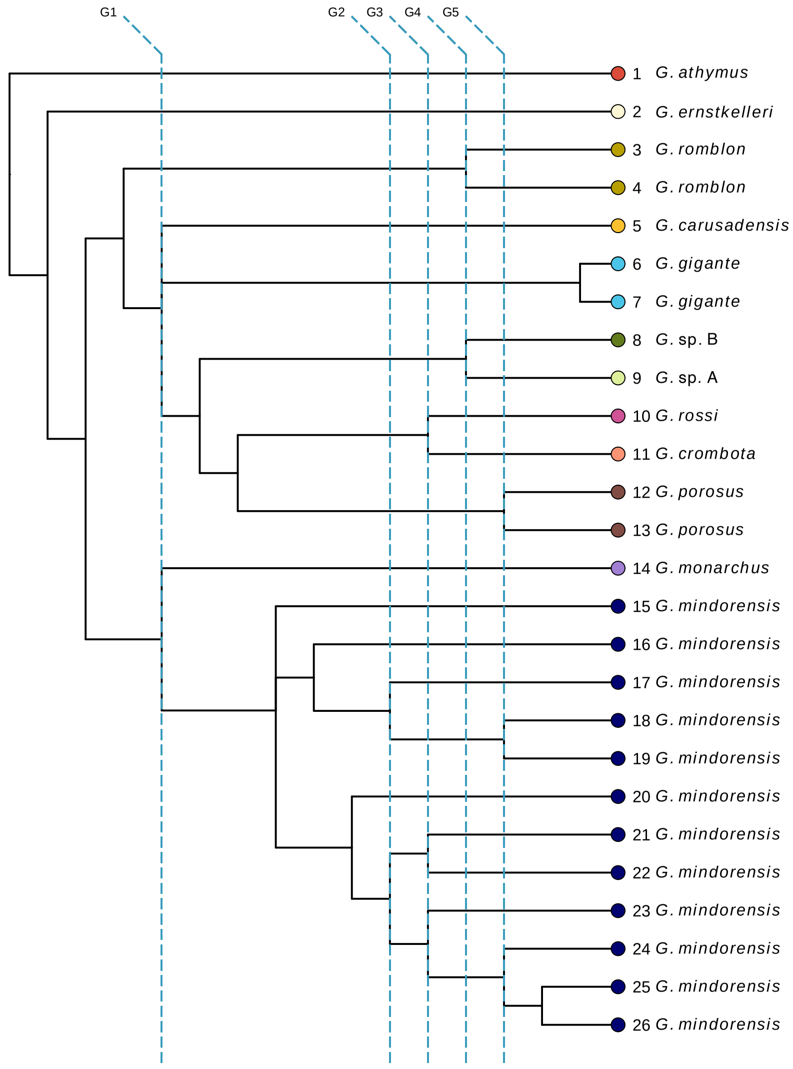

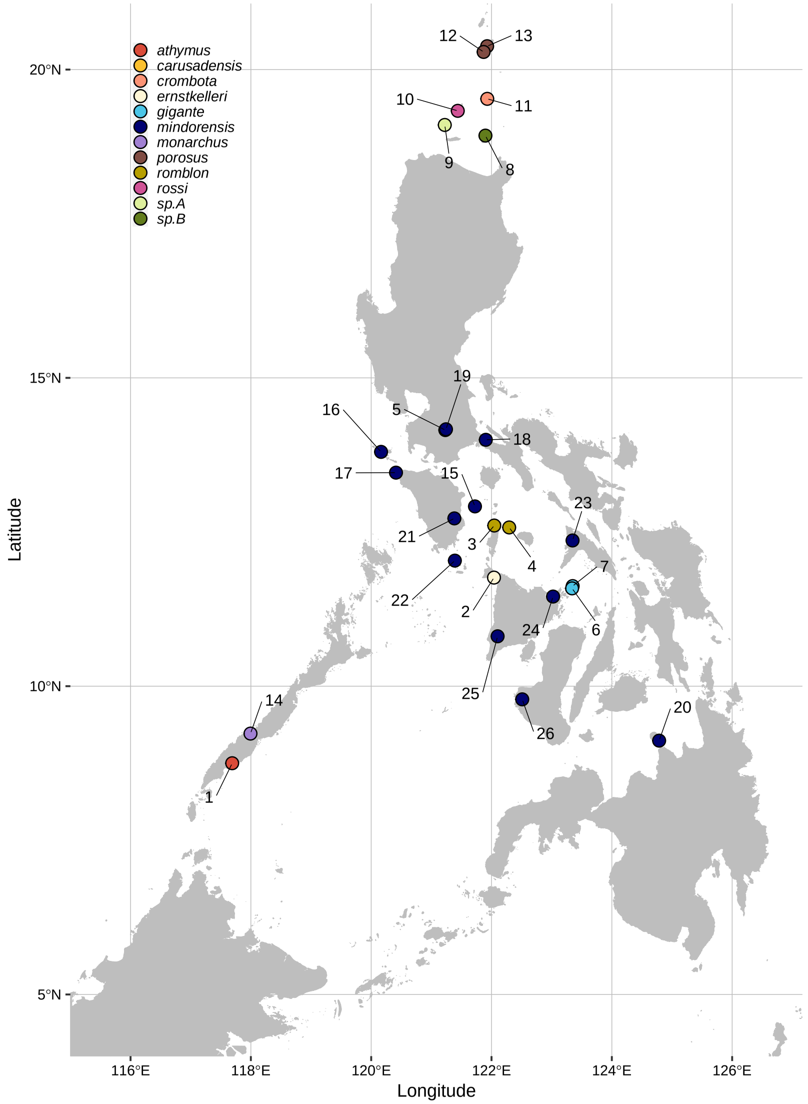

Figure S15. The maximum *a posteriori* (MAP) topology from Figure 6C for *Gekko* shown without branch lengths to make it clearer which clades are involved. Shared divergences indicated by dashed lines, with labels shown along the top that correspond to rows in Table S3, where the divergences are summarized. Created using ggplot2 (v3.3.5; Wickham, 2016), ggtree (v3.1.0; Yu et al., 2017), treeio (v1.17.0; Wang et al., 2019), cowplot (v1.1.1; Wilke, 2020), and ggrepel (v0.9.1; Slowikowski, 2020). Link to nexus-formatted annotated MAP tree: *Gekko*.

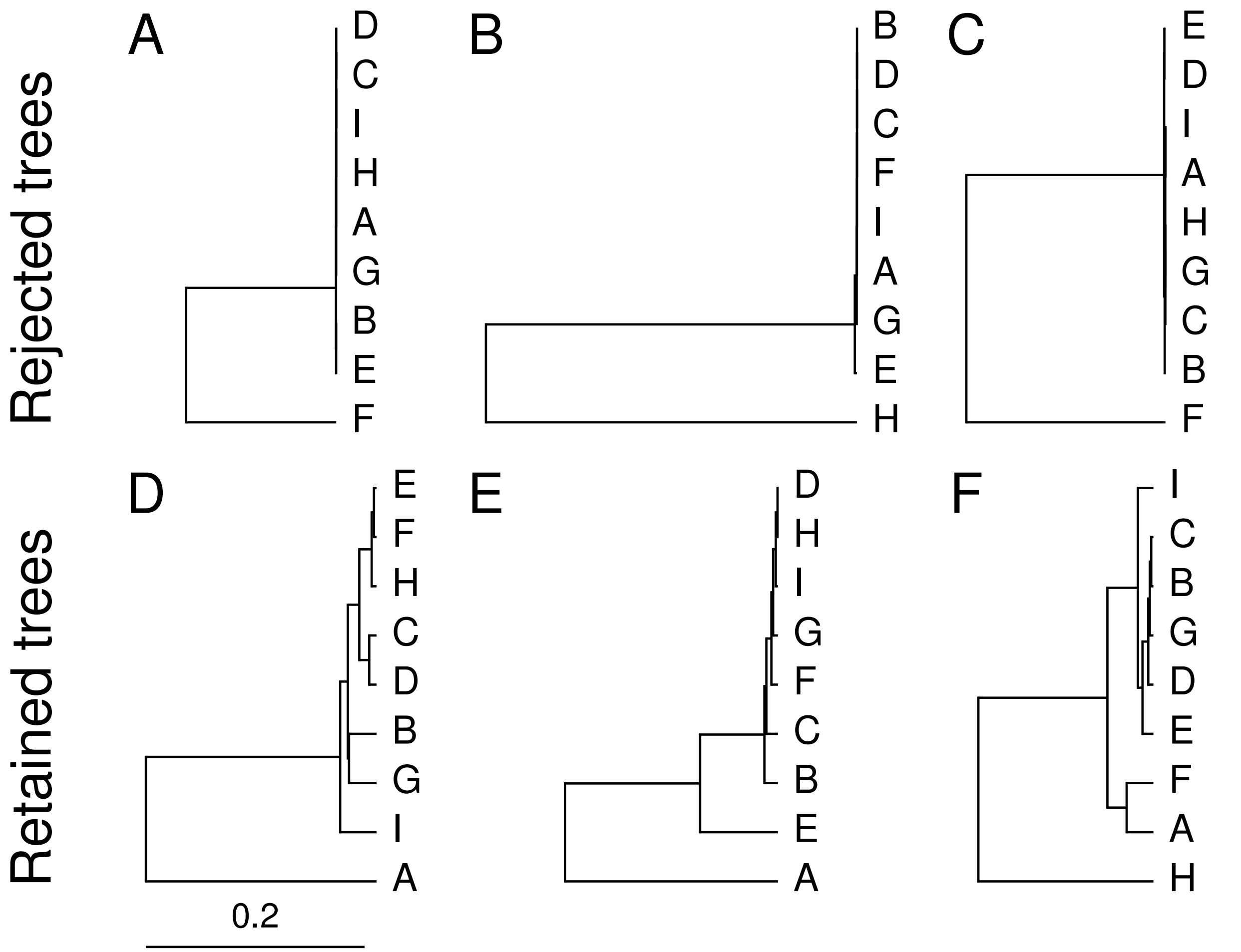

Figure S16. Examples of trees (all with 8 independent, bifurcating divergences) rejected (top) and retained (bottom) when a minimum threshold of 0.001 substitutions per site between divergence times is applied to trees randomly sampled from the prior distribution of the M_{IB} model. Trees plotted using Gram (Version 4.0.0, Commit 02286362; Foster, 2018) and the P4 phylogenetic toolkit (Version 1.4, Commit d9c8d1b1; Foster, 2004).

2 Tables

Table S1. The data for all *Cyrtodactylus* samples included in our phylogenetic analyses are included in a tab-delimited text file available from the project repository and archived on Zenodo (Oaks and Wood, Jr., 2021): https://raw.githubusercontent.com/phyletica/gekgo/master/phycoeval-msg-assemblies/ipyrad-assemblies/sample-data/Cyrt_localities.tsv. Below is a summary of the data from each sample.

Tip label	Catalog number	Species	Country	Island
1	KU 309330	*Cyrtodactylus redimiculus*	Philippines	Palawan
2	KU 309320	*Cyrtodactylus tautbatorum*	Philippines	Palawan
3	KU 319844	*Cyrtodactylus jambangan*	Philippines	Mindanao
4	KU 309365	*Cyrtodactylus annulatus*	Philippines	Camiguin Sur
5	KU 305569	*Cyrtodactylus annulatus*	Philippines	Cebu
6	KU 324366	*Cyrtodactylus annulatus*	Philippines	Bohol
7	KU 310102	*Cyrtodactylus mamanwa*	Philippines	Dinagat
8	KU 310798	*Cyrtodactylus sumuroi*	Philippines	Samar
9	KU 326522	*Cyrtodactylus gubaot*	Philippines	Leyte
10	KU 320351	*Cyrtodactylus philippinicus*	Philippines	Lubang
11	KU 315340	*Cyrtodactylus philippinicus*	Philippines	Tablas
12	KU 302635	*Cyrtodactylus philippinicus*	Philippines	Sibuyan
13	KU 305431	*Cyrtodactylus philippinicus*	Philippines	Mindoro
14	KU 313820	*Cyrtodactylus philippinicus*	Philippines	Luzon
15	KU 324376	*Cyrtodactylus philippinicus*	Philippines	Negros
16	KU 302639	*Cyrtodactylus philippinicus*	Philippines	Panay
17	KU 306612	*Cyrtodactylus philippinicus*	Philippines	Mindoro
18	KU 328804	*Cyrtodactylus philippinicus*	Philippines	Luzon
19	KU 323191	*Cyrtodactylus philippinicus*	Philippines	Luzon
20	KU 307451	*Cyrtodactylus philippinicus*	Philippines	Polillo
21	KU 330798	*Cyrtodactylus philippinicus*	Philippines	Luzon
22	KU 329351	*Cyrtodactylus philippinicus*	Philippines	Luzon
23	KU 330195	*Cyrtodactylus philippinicus*	Philippines	Luzon
24	KU 304784	*Cyrtodactylus philippinicus*	Philippines	Babuyan Claro
25	KU 325814	*Cyrtodactylus philippinicus*	Philippines	Luzon
26	KU 329534	*Cyrtodactylus philippinicus*	Philippines	Luzon
27	KU 304634	*Cyrtodactylus philippinicus*	Philippines	Camiguin Norte

Table S2. The data for all *Gekko* samples included in our phylogenetic analyses are included in a tab-delimited text file available from the project repository and archived on Zenodo (Oaks and Wood, Jr., 2021): `https://raw.githubusercontent.com/phyletica/gekgo/master/phycoeval-msg-assemblies/ipyrad-assemblies/sample-data/Gekko_localities.tsv`. Below is a summary of the data from each sample.

Tip label	Catalog number	Species	Country	Island
1	KU 309331	*Gekko athymus*	Philippines	Palawan
2	KU 300200	*Gekko ernstkelleri*	Philippines	Panay
3	KU 315348	*Gekko romblon*	Philippines	Tablas
4	KU 302738	*Gekko romblon*	Philippines	Romblon
5	KU 328372	*Gekko carusadensis*	Philippines	Luzon
6	KU 302718	*Gekko gigante*	Philippines	South Gigante
7	KU 302716	*Gekko gigante*	Philippines	North Gigante
8	KU 304673	*Gekko* sp. b	Philippines	Camiguin Norte
9	KU 307051	*Gekko* sp. a	Philippines	Dalupiri
10	KU 304935	*Gekko rossi*	Philippines	Calayan
11	KU 304848	*Gekko crombota*	Philippines	Babuyan Claro
12	KU 313977	*Gekko porosus*	Philippines	Sabtang
13	KU 313983	*Gekko porosus*	Philippines	Batan
14	KU 309295	*Gekko monarchus*	Philippines	Palawan
15	KU 302734	*Gekko mindorensis*	Philippines	Maestre De Campo
16	KU 320395	*Gekko mindorensis*	Philippines	Lubang
17	KU 308552	*Gekko mindorensis*	Philippines	Mindoro
18	KU 328822	*Gekko mindorensis*	Philippines	Luzon
19	KU 330781	*Gekko mindorensis*	Philippines	Luzon
20	KU 302684	*Gekko mindorensis*	Philippines	Camiguin Sur
21	KU 302669	*Gekko mindorensis*	Philippines	Mindoro
22	KU 302679	*Gekko mindorensis*	Philippines	Caluya
23	KU 302728	*Gekko mindorensis*	Philippines	Masbate
24	KU 302731	*Gekko mindorensis*	Philippines	Panay
25	KU 307064	*Gekko mindorensis*	Philippines	Panay
26	CDSGS 40	*Gekko mindorensis*	Philippines	Negros

Table S3. Summary of shared divergences in the maximum *a posteriori* (MAP) phylogeny estimated under the generalized tree model for *Cyrtodactylus* (Figure 6A) and *Gekko* (Figure 6C). See Figures S14 & S15 for the shared divergence labels.

Shared divergence label	Number of nodes	Posterior prob.	Mean time	Time 95% HPDI
C1	3	0.569	3.163	3.075–3.255
C2	2	0.894	2.254	2.151–2.361
C3	2	0.897	1.524	1.435–1.610
C4	2	0.449	1.195	1.112–1.281
C5	3	0.303	0.957	0.878–1.041
G1	2	0.812	10.012	9.634–10.409
G2	2	0.354	1.514	1.398–1.621
G3	3	0.192	1.228	1.123–1.343
G4	2	0.552	0.844	0.725–0.978
G5	3	0.283	0.496	0.409–0.588

Table S4. Convergence statistics we calculated with `sumphycoeval` from MCMC samples collected with `phycoeval`. We ran each MCMC chain for 15,000 generations, sampling every 10 generations.

Summary	*Cyrtodactylus*	*Gekko*
Number of chains	25	25
Samples skipped per chain	101	101
Samples retained per chain	1400	1400
Total samples	35000	35000
ASDSF	0.0027	0.00093
Root age PSRF	1.0000057	1.0001458
Root age ESS	33671.16	26844.22
Population size PSRF	1.0006948	1.0006295
Population size ESS	17542.23	13789.14

Table S5. Settings used for assembling RADseq loci for *Cyrtodactylus* and *Gekko*.

**ipyrad setting**	**Value**
assembly_method	denovo
datatype	rad
restriction_overhang	TATG,
max_low_qual_bases	5
phred_Qscore_offset	33
mindepth_statistical	6
mindepth_majrule	6
maxdepth	10000
clust_threshold	0.85
max_barcode_mismatch	0
filter_adapters	1
filter_min_trim_len	35
max_alleles_consens	2
max_Ns_consens	0.05
max_Hs_consens	0.05
min_samples_locus	4
max_SNPs_locus	0.2
max_Indels_locus	8
max_shared_Hs_locus	0.5
trim_reads	0, 0, 0, 0
trim_loci	0, 0, 0, 0

3 The generalized tree model

Let T represent a rooted, potentially multifurcating tree topology with N tips and $n(t)$ internal nodes $\boldsymbol{t} = t_1, t_2, \ldots t_{n(t)}$, where $n(t)$ can range from 1 (the "comb" tree) to $N-1$ (fully bifurcating, independent divergences). Each internal node t is assigned to one divergence time τ, which it may share with other internal nodes in the tree. We will use $\boldsymbol{\tau} = \tau_1, \ldots, \tau_{n(\tau)}$ to represent $n(\tau)$ divergence times, where $n(\tau)$ can also range from 1 to $N-1$, and every τ has at least one node assigned to it, and every node maps to a divergence time more recent than its parent (Figure S17). Note, the number of divergence times is constrained by the number of internal nodes; $n(\tau) \leq n(t)$. For convenience, we will index each τ from youngest to oldest. We assume the tree is ultrametric; all tips are at time zero, which we will denote as τ_0.

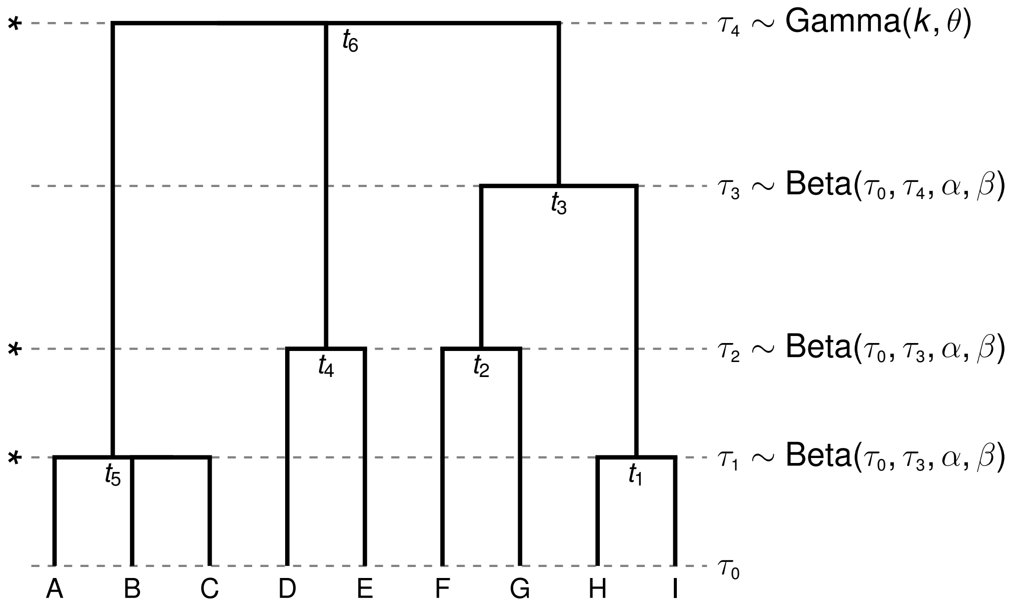

Figure S17. An illustration of the generalized tree model implemented in `ecoevolity`. The prior distributions of the divergence times are shown to the right, and "splittable" divergence times are indicated with an asterisk to the left. Figure created using Gram (Version 4.0.0, Commit 02286362; Foster, 2018) and the P4 phylogenetic toolkit (Version 1.4, Commit d9c8d1b1; Foster, 2004).

We assume all possible topologies (T) are equally probable; see Figure S1 for an example of the sample space of topologies under the generalized tree model. We assume the age of the root node follows a parametric distribution (e.g., a gamma distribution), and each of the other divergence times is beta-distributed between the present (τ_0) and the age of the youngest parent of any node mapped to the divergence time. For example, in Figure S17, the parents of the nodes mapped to τ_1 are t_3 (parent of t_1) and t_6 (parent of t_5), which are mapped to τ_3 and τ_4, respectively, the younger of which is τ_3. Thus, divergence time τ_1 in Figure S17 follows a beta distribution that is scaled to the interval between 0 and τ_3,

resulting in the prior probability density

$$f(\tau_1 \,|\, \tau_3, \alpha_\tau, \beta_\tau) = \frac{\left(\frac{\tau_1}{\tau_3}\right)^{\alpha_\tau - 1}\left(1 - \frac{\tau_1}{\tau_3}\right)^{\beta_\tau - 1}}{B(\alpha_\tau, \beta_\tau)\tau_3}. \tag{3}$$

where α_τ and β_τ are the two positive shape parameters of the beta prior probability distribution, and $B(\alpha_\tau, \beta_\tau)$ is the beta function that serves as a normalizing constant. If we use $y(\tau_i)$ to denote the divergence time of the youngest parent of any node mapped to the non-root divergence time, τ_i, we can generalize this beta prior probability density as

$$f(\tau_i \,|\, y(\tau_i), \alpha_\tau, \beta_\tau) = \frac{\left(\frac{\tau_i}{y(\tau_i)}\right)^{\alpha_\tau - 1}\left(1 - \frac{\tau_i}{y(\tau_i)}\right)^{\beta_\tau - 1}}{B(\alpha_\tau, \beta_\tau)y(\tau_i)}. \tag{4}$$

For additional flexibility, we allow a distribution to be placed on the alpha parameter of the beta distributions on the non-root divergence times (α_τ). However, we constrain $\beta_\tau = 1$, simplifying the prior probability density of each non-root divergence time to

$$f(\tau_i \,|\, y(\tau_i), \alpha_\tau) = \frac{\left(\frac{\tau_i}{y(\tau_i)}\right)^{\alpha_\tau - 1}}{B(\alpha_\tau, 1)y(\tau_i)}. \tag{5}$$

For all of our analyses presented in this paper, we constrained $\alpha_\tau = 1$, which further simplifies the prior probability of each non-root divergence time to the uniform density

$$f(\tau_i \,|\, y(\tau_i)) = \frac{1}{y(\tau_i)}. \tag{6}$$

For simplicity below, we denote the probability density of a divergence time as $f(\tau_i \,|\, y(\tau_i))$, but our work generalizes to any proper probability distributions on the divergence times, including the full beta distribution (Equation 4).

4 Approximating the posterior of the generalized tree model

We use Markov chain Monte Carlo (MCMC), specifically Metropolis-Hastings (MH) (Metropolis et al., 1953; Hastings, 1970) algorithms, to sample from the generalized tree distribution. An MH algorithm works by stochastically proposing changes to parameters of a model, and using the following rule to determine the probability of accepting these moves:

$$\text{Acceptance probability} = \min\left\{1, \begin{pmatrix}\text{likelihood}\\\text{ratio}\end{pmatrix} \times \begin{pmatrix}\text{prior}\\\text{ratio}\end{pmatrix} \times \begin{pmatrix}\text{Hastings}\\\text{ratio}\end{pmatrix}\right\} \tag{7}$$

The product of the first two terms (likelihood and prior ratios) gives us the posterior density of the state of the model being proposed divided by the posterior density of the current state

of the model. The Hastings ratio corrects for asymmetry in the proposal distribution by dividing the probability density of proposing the move that would reverse the move being proposed by the probability density of the move being proposed.

Below we describe MH moves for sampling from the generalized tree distribution, and derive the prior and Hastings ratio for each. These moves can be coupled with any likelihood function to calculate the likelihood ratio and sample from the posterior distribution of generalized trees using MH (Equation 7). In Sections 4.1–4.4, we describe a pair of reversible-jump moves, "split-time" and "merge-times," for moving between trees with different numbers of divergence times. These are the core moves that allow the full space of the generalized tree distribution to be traversed and sampled. In Section 4.5, we describe moves that propose changes to the topology, but do not change the number of divergence-time parameters, nor their values. In Section 4.6, we describe a move that updates the values of divergence times without changing the topology (though we do describe an extension of this move that does update the topology).

4.1 Split-time move

To generalize the space of trees, we introduce two reversible-jump moves: "split-time" and "merge-times." When a reversible-jump move is to be attempted, the merge-times or split-time move is chosen with probability 0.5, except for two special cases:

1. If the current state of the chain is the most general tree model ($n(\tau) = N - 1$), then the merge-times move is chosen with probability 1.
2. If the current state of the chain is the "comb" tree ($n(\tau) = 1$), then the split-time move is chosen with probability 1.

The basic idea is to randomly divide a "splittable" divergence time into two nonempty sets of nodes, and assign one of the sets to a new, more recent divergence time. A divergence time is considered splittable if it has (1) more than one node mapped to it, or (2) a single multifurcating node mapped to it. For example, in Figure S17, divergence times τ_1, τ_2, and τ_4 are splittable. The first step is to randomly choose a divergence time, τ_i, from among the splittable divergence times. After dividing τ_i into two sets of nodes, we need to randomly select a time more recent than τ_i to assign one of the sets.

4.1.1 Drawing the new divergence time

To get the new, proposed divergence time, we randomly draw a new divergence time between τ_{i-1} and τ_i from a proposal distribution, where

$$g_\tau(\tau' \mid \tau_i, \tau_{i-1}) \tag{8}$$

is the conditional probability density of proposing the new time τ' given the times from the current values of τ_i and τ_{i-1}. In our implementation, we use a beta probability distribution

scaled and shifted to the interval τ_{i-1}–τ_i, so that the probability density of the new time is

$$g_\tau(\tau' \,|\, \tau_i, \tau_{i-1}, \alpha, \beta) = \frac{\left(\frac{\tau'-\tau_{i-1}}{\tau_i-\tau_{i-1}}\right)^{\alpha-1} \left(1 - \frac{\tau'-\tau_{i-1}}{\tau_i-\tau_{i-1}}\right)^{\beta-1}}{B(\alpha,\beta)(\tau_i - \tau_{i-1})}, \tag{9}$$

where α and β are the two positive shape parameters of the beta distribution, and $B(\alpha, \beta)$ is the beta function . For generality and simplicity, we will use g_τ to denote this probability density of the proposed divergence time below.

4.1.2 Prior ratio

The prior ratio for the split-time move is

$$\frac{f(T', \boldsymbol{\tau'})}{f(T, \boldsymbol{\tau})}, \tag{10}$$

where $f(T', \boldsymbol{\tau'})$ is the prior probability of the proposed tree topology and divergence times. In our implementation, we assume that all possible tree topologies (across $n(\tau) = 1, 2, \ldots, N-1$) are equally probable *a priori*. We also assume the divergence time of the root ($\tau_{n(\tau)}$) is gamma-distributed, and each of the other divergence times is beta-distributed between the present (τ_0) and the divergence time of the youngest parent of a node mapped to τ_i, which we denote as $y(\tau_i)$ (Figure S17; Equation 4). Given these assumptions, the prior ratio becomes

$$\frac{f(\tau' \,|\, y(\tau'))f(\tau_{i-1} \,|\, y(\tau_{i-1})')}{f(\tau_{i-1} \,|\, y(\tau_{i-1}))}, \tag{11}$$

If $\tau_i = 1$ (i.e., the divergence time selected to split was the most recent divergence), then τ_{i-1} is the present, and so $f(\tau_{i-1} \,|\, y(\tau_{i-1})') = f(\tau_{i-1} \,|\, y(\tau_{i-1})) = 1$. Also, if none of the nodes assigned to τ_{i-1} has a parent assigned to the newly proposed divergence time (i.e., $y(\tau_{i-1})' \neq \tau'$), then $y(\tau_{i-1})' = y(\tau_{i-1})$; e.g., in Figure S17, if τ_2 is split, the prior probability density of τ_1 is not affected, because the youngest parent of a node mapped to τ_1 is mapped to τ_3. In both of these special cases, the prior ratio further simplifies to

$$f(\tau' \,|\, y(\tau')) \tag{12}$$

4.1.3 Hastings ratio

The probability of proposing a split-time move involves several components. First, we have to choose to split rather than merge. We will account for the probability of this toward the end of this section. Next, we randomly choose a splittable divergence time τ_i with probability $\frac{1}{n_s(\tau)}$, where $n_s(\tau)$ is the number of splittable divergence times. As described in Section 4.1.1 above, we randomly choose a new divergence time τ' more recent than τ_i with probability density g_τ.

When we divide τ_i into two sets of nodes, if any polytomies get broken up, new branches will get added to the tree. Under certain models, each of these new branches will need values

randomly drawn for parameters. For example, if using a "relaxed-clock" model, each new branch will need a substitution rate. Or, if using a multi-species coalescent model where each branch has its own effective population size, a value for this will need to be drawn. Note, this does not involve divergence-time parameters, because all nodes split from τ_i will be assigned to τ'. We will use $\boldsymbol{g}_z$ to represent the product of all the probability densities of the proposed values for the new branches. If no polytomies get broken up, or new branches created from broken polytomies do not require parameter values, then $\boldsymbol{g}_z = 1$.

We will deal with how the nodes assigned to τ_i are divided into two sets below. For now, we will use Ξ to represent the probability of the proposed division of τ_i. The probability density of the proposed split move is then

$$g(\Theta' \mid \Theta) = \frac{\boldsymbol{g}_z g_\tau \Xi}{n_s(\tau)}, \tag{13}$$

where Θ and Θ' represent the full state of the model before and after the proposed split move, respectively. The move that would exactly reverse this split move would simply entail randomly selecting the proposed divergence time from all divergence times except the root, which would then be deterministically merged with the next older divergence time. The probability of this reverse move is

$$g(\Theta \mid \Theta') = \frac{1}{n(\tau)' - 1} = \frac{1}{n(\tau)}, \tag{14}$$

where $n(\tau)$ and $n(\tau)'$ is the number of divergence times before and after the proposed split move, respectively.

The Hastings ratio for the split move is then

$$\frac{g(\Theta \mid \Theta')}{g(\Theta' \mid \Theta)} = \gamma_S \frac{n_s(\tau)}{n(\tau)\boldsymbol{g}_z g_\tau \Xi}, \tag{15}$$

where γ_S represents the probability of choosing to merge in the reverse move divided by the probability of choosing to split in the forward move, which is

$$\gamma_S = \begin{cases} 0.5 & \text{if current tree is the ``comb'' and proposed tree has } n(\tau) < N - 1 \\ 2.0 & \text{if proposed tree has } n(\tau) = N - 1 \text{ and the current tree is not the ``comb''} \\ 1.0 & \text{otherwise.} \end{cases} \tag{16}$$

When we are working with a tree with more than three tips, the first case occurs whenever the current tree is the comb tree ($n(\tau) = 1$), and the second case occurs whenever the proposed tree has no shared divergences nor multifurcations ($n(\tau) = N - 1$). However, for full generality we need to include the second condition in both of these cases to account for the situation where we are working with a tree with only three tips.

4.2 Merge-times move

In the "merge-times" move, we randomly choose τ_x from one of the $n(\tau) - 1$ non-root divergence times. Then, we merge τ_x with the next older divergence time, τ_{x+1}. This will create shared divergence times among nodes and/or multifurcating nodes. We will use τ'_{x+1} to refer to the newly merged divergence time proposed by the move.

4.2.1 Prior ratio

Generally, the prior ratio for the merge-times move is the same as Equation 10. Assuming (1) all topologies are equally probable, (2) the divergence time of the root ($\tau_{n(\tau)}$) is gamma-distributed, and (3) each of the other divergence times is beta-distributed between the present (τ_0) and the age of the youngest parent to the nodes mapped to the divergence time (Figure S17; Equation 4), the prior ratio becomes

$$\frac{f(\tau'_{x+1} \,|\, y(\tau'_{x+1}))}{f(\tau_x \,|\, y(\tau_x)) f(\tau_{x+1} \,|\, y(\tau_{x+1}))}. \tag{17}$$

If τ_{x+1} is the root of the tree, then $f(\tau'_{x+1} \,|\, y(\tau'_{x+1})) = f(\tau'_{x+1}) = f(\tau_{x+1} \,|\, y(\tau_{x+1})) = f(\tau_{x+1})$, and this probability density is given by the gamma prior distribution on the divergence time of the root (Figure S17).

4.2.2 Hastings ratio

The probability of the forward merge-times move is simply

$$g(\Theta' \,|\, \Theta) = \frac{1}{n(\tau) - 1} = \frac{1}{n(\tau)'} \tag{18}$$

where $n(\tau)$ and $n(\tau)'$ is the number of divergence times before and after the proposed merge move, respectively.

Borrowing from Equation 13, the probability density of the split move that would exactly reverse the proposed merge move is

$$g(\Theta \,|\, \Theta') = \frac{\boldsymbol{g}_z g_\tau \Xi}{n_s(\tau)'}, \tag{19}$$

where $n_s(\tau)'$ is the number of splittable divergence times *after* the proposed merge-times move.

The Hastings ratio for the merge move is then

$$\frac{g(\Theta \,|\, \Theta')}{g(\Theta' \,|\, \Theta)} = \gamma_M \frac{n(\tau)' \boldsymbol{g}_z g_\tau \Xi}{n_s(\tau)'}, \tag{20}$$

where γ_M represents the probability of choosing to split in the reverse move divided by the

probability of choosing to merge in the forward move, which is

$$\gamma_M = \begin{cases} 2.0 & \text{if proposed tree is the ``comb'' and current tree has } n(\tau) < N-1 \\ 0.5 & \text{if current tree has } n(\tau) = N-1 \text{ and the proposed tree is not the ``comb''} \\ 1.0 & \text{otherwise} \end{cases} \tag{21}$$

4.3 Expanding Ξ

Up to this point, we have not dealt with how, during the split-time move, we divide the nodes mapped to τ_i into two sets, one of which gets assigned to the new divergence time drawn between τ_{i-1} and τ_i. This has to be done with care to ensure that every possible configuration of two divergence times derived from the nodes assigned to τ_i can be proposed, such that it properly balances the reverse merge-times move. As above, we use Ξ to represent the probability of the proposed division of τ_i's nodes.

In the next two sections, we show how this is done for two special cases. The first special case illustrates how we first choose which nodes currently mapped to τ_i will get moved to the new divergence time. The second special case shows how we handle any multifurcating nodes that have been chosen to be moved to the new divergence time. In the third section, we build on these special cases to show a general solution for Ξ.

4.3.1 The case of all bifurcating nodes mapped to τ_i

We will use $n(t \mapsto \tau_i)$ to represent the number of nodes mapped to τ_i. If all $n(t \mapsto \tau_i)$ nodes mapped to τ_i are bifurcating, we randomly divide these nodes into two non-empty sets and then randomly choose one of the two sets of nodes to move to the new divergence time. For example, this would be the case if τ_2 is chosen to split from the tree shown in Figure S17.

The number of ways $n(t \mapsto \tau_i)$ can be divided into two non-empty subsets is given by the Stirling number of the second kind, which we denote as $S_2(n(t \mapsto \tau_i), 2)$. We uniformly choose among these, such that the probability of randomly selecting any set partition of the $n(t \mapsto \tau_i)$ nodes mapped to τ_i is $\frac{1}{n(t \mapsto \tau_i)}$. After partitioning the nodes into two sets, there is a 1/2 probability of choosing one set to move to the new, more recent divergence time. Thus, *when all of the nodes mapped to τ_i are bifurcating* the probability of each possible splitting of τ_i is

$$\Xi = \frac{1}{2 \times S_2(n(t \mapsto \tau_i), 2)} = \frac{1}{2^{n(t \mapsto \tau_i)} - 2}. \tag{22}$$

4.3.2 The case of a single polytomy mapped to τ_i

Next, let's consider another special case where the number of nodes mapped to τ_i is one (i.e., a single polytomy). For example, this would be the case if τ_4 is chosen to split from the tree shown in Figure S17. In this case, we randomly resolve the polytomy, by randomly (uniformly) choosing a set partition of the descending branches into non-empty subsets. Any subsets with only one branch remain attached to the original polytomy node,

while each subset with multiple branches get split off to form a new node (clade) that descends from the original polytomy node. These new nodes are assigned to the new, more recent divergence time τ'. The number of ways to partition the descending branches of the polytomy are thus $B_b - 2$, where B_b is the Bell number (Bell, 1934)—the number of possible set partitions of the b branches descending from the polytomy. We have to subtract 2 from B_b, because we do not allow the two "extreme" set partitions with one or b subsets. The former would move the whole polytomy to the new divergence time, and the latter would leave the polytomy as is. Neither of these scenarios adds a dimension (divergence time) to the model. We avoid these two scenarios using rejection so that the remaining partitions of the b descending branches are chosen uniformly. Thus, *when only a single polytomy is mapped to* τ_i the probability of each possible splitting of τ_i is

$$\Xi = \frac{1}{B_b - 2}. \tag{23}$$

4.3.3 The case when multiple nodes, including at least one polytomy, are mapped to τ_i

When multiple nodes are mapped to τ_i, and at least one is a polytomy, we need to do some more accounting to ensure that we can reach every possible arrangement of two divergence times that can be merged to form the current configuration of nodes mapped to τ_i. Similar to the case with all bifurcating nodes, we will first divide the $n(t \mapsto \tau_i)$ nodes mapped to τ_i into two subsets and randomly choose one of these subsets to move to the proposed, more recent divergence time, τ'. For each multifurcating node that ends up in the subset to be moved to τ' (if any), we need to either break up the polytomy, as we did in the case of the single-polytomy case above, or move the entire polytomy to τ'.

Unlike in the case of only bifurcating nodes mapped to τ_i, when we partition the $n(t \mapsto \tau_i)$ nodes mapped to τ_i into two sets, we must allow for the case where all $n(t \mapsto \tau_i)$ end up in the set to move to τ'. This is because, if any of the polytomy nodes get broken up, they will leave at least one node at τ_i, and the dimension of the model will change (i.e., the number of divergence times will increase by one). So, we have to allow an empty subset when we randomly partition the $n(t \mapsto \tau_i)$ into two subsets. However, we cannot allow the empty subset to be chosen to move to τ'. There are $S_2(n(t \mapsto \tau_i), 2) + 1$ ways to partition the $n(t \mapsto \tau_i)$ nodes mapped to τ_i into two subsets if we allow the set partition with one empty subset. For each of these, there are two ways to choose the subset to move to τ', and of all of these, there is one scenario we will reject: if the empty set gets selected to move to τ'. Thus, there are

$$(2(S_2(n(t \mapsto \tau_i), 2) + 1)) - 1 = (2S_2(n(t \mapsto \tau_i), 2)) + 1 = 2^{n(t \mapsto \tau_i)} - 1 \tag{24}$$

ways to choose a subset of the nodes assigned to τ_i for moving to the new divergence time, and the probability of each is

$$\frac{1}{2^{n(t \mapsto \tau_i)} - 1}. \tag{25}$$

For each polytomy mapped to τ_i that ends up in the set of nodes to move to τ' (if any), we randomly choose one of the B_b possible set partitions of the b branches descending from the polytomy. However, we will reject the set partition with b subsets (i.e., all branches end up in their own subset). We reject this, because no subclades get broken off from the polytomy to move to τ', and this scenario is already taken into account by the polytomy node not ending up in the set of nodes to move to τ' in the first place. However, we need to allow the scenario where all b branches descending from a polytomy get assigned to a single set, which results in the entire polytomy node getting moved to τ', as long as at least one node remains assigned to τ_i (we will handle this in a bit). Thus, for each polytomy in the set of nodes to be moved to τ', there are $B_b - 1$ ways to move it. Using $n_p(t \Rightarrow \tau')$ to represent the number of polytomies in the subset of nodes to be moved to τ', the total number of ways these polytomies can be moved to τ' is

$$\Phi = \prod_{x=1}^{n_p(t \Rightarrow \tau')} \left(B_{b_x} - 1\right), \tag{26}$$

and the probability of each is equal to

$$\prod_{x=1}^{n_p(t \Rightarrow \tau')} \frac{1}{(B_{b_x} - 1)} = \frac{1}{\prod_{x=1}^{n_p(t \Rightarrow \tau')} (B_{b_x} - 1)} = \frac{1}{\Phi}. \tag{27}$$

If no polytomies end up in the subset of nodes to move to τ', then $\Phi = 1$.

However, if all $n(t \mapsto \tau_i)$ nodes mapped to τ_i end up in the set of nodes to be moved to τ', we need to reject the case where none of the polytomy nodes gets broken up (i.e., for every polytomy, all the descending branches get partitioned into a single set), because no nodes would remain assigned to τ_i, and the move would simplify to changing the value of τ_i. Thus, if all $n(t \mapsto \tau_i)$ nodes mapped to τ_i end up in the set of nodes to move to τ', the total number of ways all $n_p(t \Rightarrow \tau')$ polytomies can be moved to τ' is

$$\left(\prod_{x=1}^{n_p(t \Rightarrow \tau')} \left(B_{b_x} - 1\right)\right) - 1 = \Phi - 1, \tag{28}$$

and the probability of each is equal to

$$\frac{1}{\Phi - 1}. \tag{29}$$

Given all of this, the probability of choosing a subset of nodes from τ_i to move to the new divergence time across all possible cases is

$$\Xi = \begin{cases} \frac{1}{2^{n(t \mapsto \tau_i)} - 2} & \text{if no polytomies mapped to } \tau_i \\ \frac{1}{\left(2^{n(t \mapsto \tau_i)} - 1\right)(\Phi - 1)} & \text{if } \geq 1 \text{ polytomy nodes mapped to } \tau_i \text{, and all } n(t \mapsto \tau_i) \text{ assigned to move set} \\ \frac{1}{\left(2^{n(t \mapsto \tau_i)} - 1\right)\Phi} & \text{otherwise.} \end{cases}$$

(30)

Notice, the case of $n(t \mapsto \tau_i) = 1$ (i.e., only a single polytomy node mapped to τ_i) is simply a special case of the second condition above, where all the nodes assigned to τ_i end up in the set to move to the new divergence time, including at least one polytomy.

4.4 Validation of Split-time and Merge-times moves

To validate the split-time/merge-times moves, we used them to sample from the prior distribution of trees with 5, 6, and 7 leaves. If working correctly, we should sample all $n(T)$ tree topologies with an equal frequency of $\frac{1}{n(T)}$. If we collect $\mathcal{N}$ MCMC samples from the prior distribution, the number of times a topology is sampled should be approximately distributed as Binomial$(n = \mathcal{N}, p = \frac{1}{n(T)})$; i.e., binomially distributed where the number of "trials" is equal to the number of samples, and the probability of sampling each topology is $\frac{1}{n(T)}$. We found a close match between the number or times each tree was sampled by our reversible-jump MCMC chain and the expected number, and failed to reject the expected binomial distribution using χ^2 goodness-of-fit test (Figure S18; $p = 0.742$, 0.464, and 0.172 for the test with a 5, 6, and 7-leaved tree).

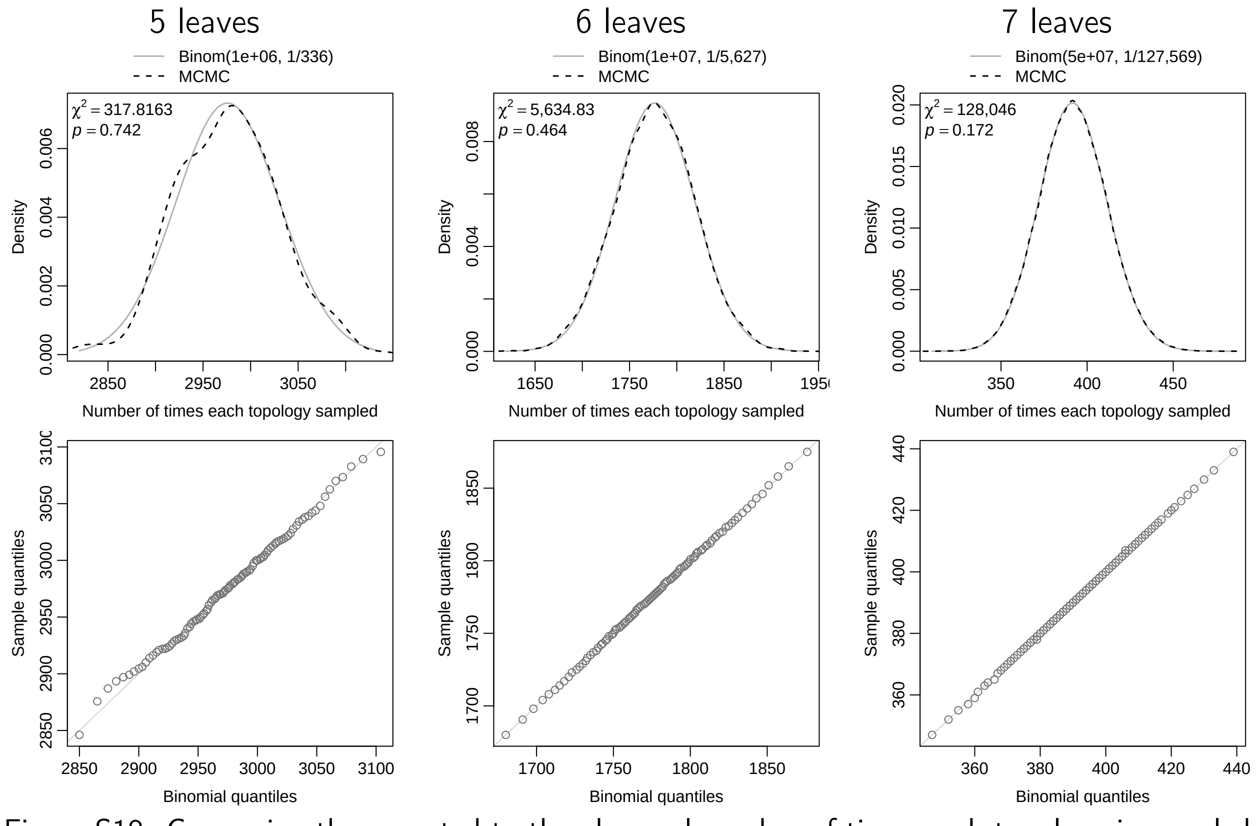

Figure S18. Comparing the expected to the observed number of times each topology is sampled by an MCMC chain using our split-time and merge-times moves. Under our generalized tree distribution, how often each topology is sampled should follow a Binomial$(n = \mathcal{N}, p = \frac{1}{n(T)})$ distribution, where $\mathcal{N}$ is the total number of MCMC samples.

4.5 Nested-neighbor-node-swap move

The split-time and merge-times moves can sample all of the space of the generalized tree distribution (assuming the age of the root node is fixed). However, we implemented additional topology moves that do not jump between tree models with different numbers of divergence times.

The goal of this move is to change the topology without changing the number or timing of divergences. We start by randomly picking a non-root divergence time, τ_i, with probability $\frac{1}{n(\tau)-1}$. Next, we find the divergence time, τ_j, that contains the node that is the youngest parent of nodes mapped to τ_i. We then randomly pick one of the nodes mapped to τ_j that has children mapped to τ_i, we will call it t_a. Each child of t_a that is mapped to τ_i will randomly contribute one of its children to a "swap pool" of nodes. If t_a has children that are *not* mapped to τ_i, we randomly pick one of these children and add it to the swap pool *if* it is younger than τ_i. If the selected child of t_a is older than τ_i, we randomly sample one of its children and continue to do so until we have chosen a descendant node that is younger than τ_i, which we then add to the swap pool. Lastly, we randomly pick two nodes from the swap pool and we swap their parents.

After the proposed move, the structure of the tree rootward of the swapped nodes is the same. Because of this, the move that would reverse the proposed move would be equally probable; it would involve (1) choosing the same non-root divergence time τ_i, (2) choosing the same t_a, (3) choosing the children that swapped parents in the forward move to enter the swap pool, and (4) picking the nodes that swapped parents in the forward move from the pool and swapping their parents back. In #3, all the parent nodes involved have the same number of children as before the proposed forward move, and so the probability of the reverse move will be equal. As a result, the Hastings ratio for the move is 1.

For example, for the tree in Figure S17 if we randomly selected τ_1, the divergence containing the youngest parent of the nodes mapped to τ_1 is τ_3. Divergence time τ_3 only has one node that is a parent of nodes assigned to τ_1, which is t_3. Node t_3 only has one child mapped to τ_1 (t_1), which will randomly contribute one of its children, Leaf H or I, to the swap pool. Node t_3 also has children that are not mapped to τ_1, one child t_2 that is mapped to τ_2. Node t_2 is considered for the swap pool, but it is too old (it is older than τ_1 and thus could not become a child of t_1. So, we randomly consider one the children of t_2, Leaf F or G, for the swap pool, either of which is young enough to be added to the swap pool. Next, we randomly choose two nodes from the swap pool, which has exactly two nodes in this case, a child of t_1 (Leaf H or I) and t_2 (Leaf F or G), and these nodes swap parents. If we assume that Leaves G and H were swapped, it is clear that the probability of the reverse move that would swap them back is equally probable.

We also implemented variations of this move that make larger changes to the tree topology. For example, we can perform the swap for all of the nodes mapped to τ_j that have children mapped to τ_i (instead of randomly choosing one of them). Another option is to randomly permute the parents of all of the nodes in the swap pool, rather than swap the parents of just two of the nodes. By chance, when doing this permutation of the nodes in the swap pool, it is possible to end up with the same topology we started with. To avoid proposing the same state, we iteratively permute the parents of the nodes in the swap pool until we have a new topology (the parents of at least some of the nodes in the swap pool

have changed). Just like with the swap move, this permutation move can be performed on one randomly selected node of τ_j that has children mapped to τ_i, or to all of them.

4.5.1 Validation of move

To validate this move, we used it to sample from a uniform distribution over the topologies of a 6-leaved bifurcating tree. There are 945 topologies for a rooted, bifurcating tree (Felsenstein, 1978). If the move is working correctly, the number of times we sample each of them should follow a Binomial($n = \mathcal{N}, p = \frac{1}{945}$) distribution, where $\mathcal{N}$ is the total number of MCMC samples. From an MCMC sample of 100,000 trees, we found a close match to this expected distribution, and were unable to reject it using a χ^2 goodness-of-fit test (Figure S19; $p = 0.51$).

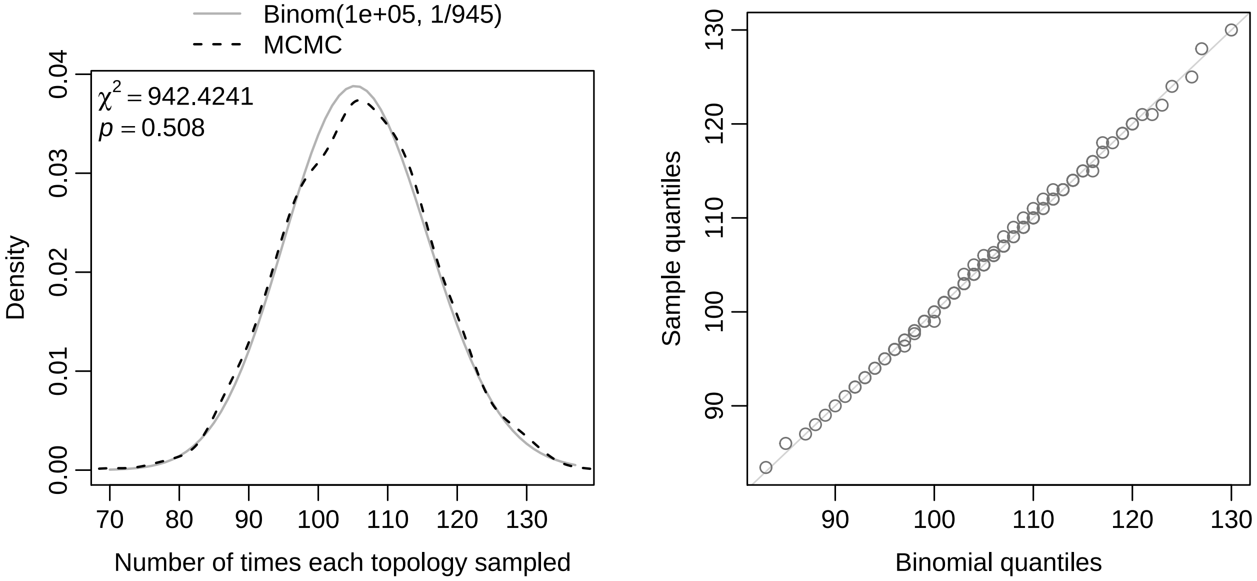

Figure S19. Comparing the expected to the observed number of times each topology of a rooted, 6-leaved, bifurcating tree is sampled by our nested-neighbor-node-swap move. Our MCMC sample of 100,000 trees closely matched the expected Binomial($n = 100,000, p = \frac{1}{945}$) distribution.

4.6 Divergence time slide bump move

To begin this move, we randomly pick one of the divergence times, τ_i. Next, we draw a uniform deviate, $u \sim$ Uniform($-\lambda, \lambda$), where λ is a tuning parameter that can be adjusted to improve the acceptance rate of the proposal. Then, we get a new divergence time value by $\tau_i e^u$. We will index our randomly selected divergence time, τ_i, as τ_1. We then use $\tau_1, \tau_2, \ldots, \tau_n$ to represent the selected time, τ_1, and all the divergence times between τ_1 and $\tau_1 e^u$ that contain nodes ancestral or descendant to the nodes mapped to τ_1. Note, that incrementing indices count younger or older divergence times, depending on whether $\tau_i e^u < \tau_1$ or $\tau_i e^u > \tau_1$, respectively.

The simplest case is that we do not have any intervening divergence times, and so we only have τ_1. This will happen when $\tau_1 e^u$ is older than the oldest node that is a child of the

nodes mapped to τ_i and younger than the youngest node that is parent of the nodes mapped to τ_i In that case, we propose a new time to which to slide τ_1 as

$$\tau_1' = \tau_1 e^u. \tag{31}$$

To reverse this move (slide τ_1 back) would be

$$\tau_1 = \tau_1' e^{u'}. \tag{32}$$

To solve for the uniform deviate that would exactly reverse the move (u'), we take the log of Equation 32 and solve for u',

$$\begin{aligned} ln(\tau_1) &= ln(\tau_1') + ln(e^{u'}) \\ ln(\tau_1) &= ln(\tau_1') + u' \\ u' &= ln(\tau_1) - ln(\tau_1') \\ u' &= ln(\tau_1) - ln(\tau_1 e^u) \\ u' &= ln(\tau_1) - ln(\tau_1) - u \\ u' &= -u. \end{aligned} \tag{33}$$

To get the Hastings ratio for this move, we use the formula of Green (1995),

$$\text{Hastings ratio} = \frac{g'(u')}{g(u)} |\det(J)|, \tag{34}$$

which is the ratio of the probability of drawing the random deviate that would reverse the proposed move to the probability of drawing the random deviate of the proposed move, multiplied by the absolute value of the determinant of a Jacobian matrix. Because the forward and reverse random deviates are uniform, $\frac{g'(u')}{g(u)} = 1$, and the Hastings ratio reduces to just the Jacobian term,

$$\begin{aligned} J &= \begin{bmatrix} \frac{\partial \tau_1'}{\partial \tau_1} & \frac{\partial \tau_1'}{\partial u} \\ \frac{\partial u'}{\partial \tau_1} & \frac{\partial u'}{\partial u} \end{bmatrix} \\ &= \begin{bmatrix} e^u & \tau_1 e^u \\ 0 & -1 \end{bmatrix} \\ \det(J) &= -e^u \\ |\det(J)| &= |-e^u| = e^u = \text{Hastings ratio}. \end{aligned} \tag{35}$$

In the next simplest case, there is one intervening divergence time τ_2. In this case, τ_1 will slide to τ_2 and "bump" it to the new time $\tau_1 e^u$. More formally, the move will be

$$\tau_2' = \tau_1 e^u$$

$$\tau_1' = \tau_2.$$

Again, the uniform deviate that would exactly reverse this move would be

$$u' = -u$$

and the reverse move would be

$$\tau_2 = \tau_1'$$

$$\tau_1 = \tau_2' e^{u'}.$$

Again, the Hastings ratio reduces to just the Jacobian term,

$$\begin{aligned} J &= \begin{bmatrix} \frac{\partial \tau_2'}{\partial \tau_2} & \frac{\partial \tau_2'}{\partial \tau_1} & \frac{\partial \tau_2'}{\partial u} \\ \frac{\partial \tau_1'}{\partial \tau_2} & \frac{\partial \tau_1'}{\partial \tau_1} & \frac{\partial \tau_1'}{\partial u} \\ \frac{\partial u'}{\partial \tau_2} & \frac{\partial u'}{\partial \tau_1} & \frac{\partial u'}{\partial u} \end{bmatrix} \\ &= \begin{bmatrix} 0 & e^u & \tau_1 e^u \\ 1 & 0 & 0 \\ 0 & 0 & -1 \end{bmatrix} \\ \det(J) &= 0 \begin{bmatrix} 0 & 0 \\ 0 & -1 \end{bmatrix} - e^u \begin{bmatrix} 1 & 0 \\ 0 & -1 \end{bmatrix} + \tau_1 e^u \begin{bmatrix} 1 & 0 \\ 0 & 0 \end{bmatrix} \\ \det(J) &= e^u \\ |\det(J)| &= e^u = \text{Hastings ratio.} \end{aligned} \quad (36)$$

To generalize this to an arbitrary number of intervening divergence times that will be bumped, we have

$$\tau_n' = \tau_1 e^u$$

$$\tau_{n-1}' = \tau_n$$

$$\tau_2' = \tau_3$$

$$\tau_1' = \tau_2.$$

Again, the uniform deviate that would exactly reverse this move would be

$$u' = -u,$$

and the reverse move would be

$$\tau_n = \tau_{n-1}'$$

$$\tau_{n-1} = \tau_{n-2}'$$

$$\tau_2 = \tau_1'$$
$$\tau_1 = \tau_n' e^{u'}.$$

The Hastings ratio reduces to just the Jacobian term,

$$J = \begin{bmatrix} \frac{\partial \tau_n'}{\partial \tau_n} & \frac{\partial \tau_n'}{\partial \tau_{n-1}} & \cdots & \frac{\partial \tau_n'}{\partial \tau_2} & \frac{\partial \tau_n'}{\partial \tau_1} & \frac{\partial \tau_n'}{\partial u} \\ \frac{\partial \tau_{n-1}'}{\partial \tau_n} & \frac{\partial \tau_{n-1}'}{\partial \tau_{n-1}} & \cdots & \frac{\partial \tau_{n-1}'}{\partial \tau_2} & \frac{\partial \tau_{n-1}'}{\partial \tau_1} & \frac{\partial \tau_{n-1}'}{\partial u} \\ \vdots & \vdots & \ddots & \vdots & \vdots & \vdots \\ \frac{\partial \tau_2'}{\partial \tau_n} & \frac{\partial \tau_2'}{\partial \tau_{n-1}} & \cdots & \frac{\partial \tau_2'}{\partial \tau_2} & \frac{\partial \tau_2'}{\partial \tau_1} & \frac{\partial \tau_2'}{\partial u} \\ \frac{\partial \tau_1'}{\partial \tau_n} & \frac{\partial \tau_1'}{\partial \tau_{n-1}} & \cdots & \frac{\partial \tau_1'}{\partial \tau_2} & \frac{\partial \tau_1'}{\partial \tau_1} & \frac{\partial \tau_1'}{\partial u} \\ \frac{\partial u'}{\partial \tau_n} & \frac{\partial u'}{\partial \tau_{n-1}} & \cdots & \frac{\partial u'}{\partial \tau_2} & \frac{\partial u'}{\partial \tau_1} & \frac{\partial u'}{\partial u} \end{bmatrix}$$
$$= \begin{bmatrix} 0 & 0 & \cdots & 0 & e^u & \tau_1 e^u \\ 1 & 0 & \ddots & 0 & 0 & 0 \\ \vdots & \ddots & \ddots & \ddots & \vdots & \vdots \\ 0 & 0 & \ddots & 0 & 0 & 0 \\ 0 & 0 & \cdots & 1 & 0 & 0 \\ 0 & 0 & \cdots & 0 & 0 & -1 \end{bmatrix} \tag{37}$$
$$\det(J) = e^u \begin{bmatrix} 1 & 0 & \ddots & 0 & 0 \\ \vdots & \ddots & \ddots & \ddots & \vdots \\ 0 & 0 & \ddots & 0 & 0 \\ 0 & 0 & \cdots & 1 & 0 \\ 0 & 0 & \cdots & 0 & -1 \end{bmatrix} - \tau_1 e^u \begin{bmatrix} 1 & 0 & \ddots & 0 & 0 \\ \vdots & \ddots & \ddots & \ddots & \vdots \\ 0 & 0 & \ddots & 0 & 0 \\ 0 & 0 & \cdots & 1 & 0 \\ 0 & 0 & \cdots & 0 & 0 \end{bmatrix}$$
$$= -e^u$$
$$|\det(J)| = |-e^u| = e^u = \text{Hastings ratio}.$$

We avoid values of $\tau_1 e^u$ that are less than zero, by rejecting the proposed move. There is no upper limit for the move, because the root of the tree can be moved to an arbitrarily old divergence time. However, in our implementation, the prior on the divergence time of the root is different than the other divergence times, and can be much more informative. In such cases, we might be able to improve mixing and tuning of the move be excluding the root divergence time from the move. We do this by selecting only non-root divergence times, and rejecting any proposed moves where $\tau_1 e^u$ is older than the root.

4.6.1 An extension to this move

We can easily extend this move to also propose new topologies. Whenever we have a "bump" that involves a node and its children, we can propose a node swapping or permuting move described above (see the nested-neighbor-node-swap move and its extensions). Because we are sliding the nodes to take the position of the nodes they bump, the swap or permute moves are simplified a bit. We do not have to worry about τ_j contributing a child that is older than its potential new parents, so we never need to randomly choose descendants until we find a node that is younger than τ_i. We implemented this move, but jointly proposing changes to continuous divergence time parameters and changing the topology might lead to poor acceptance rates (Yang, 2014), so using separate moves to update divergence times and the topology is likely a better strategy.

4.6.2 Validation of this move

We used this move to sample from the prior distribution to ensure that the distribution of sampled divergence times matched the gamma-distributed prior we placed on the root age and the beta priors we placed on all other divergence times. To validate the extension of this move that also incorporates node swapping when nodes "bump," we used it to sample from a uniform distribution over the topologies of a 6-leaved bifurcating tree. If the move is working correctly, the number of times we sample each of the 945 topologies should be follow a Binomial($n = \mathcal{N}, p = \frac{1}{945}$) distribution, where $\mathcal{N}$ is the total number of MCMC samples. We found a close match between our samples and this expected distribution, and could not reject it using a χ^2 goodness-of-fit test (Figure S20; $p = 0.165$,).

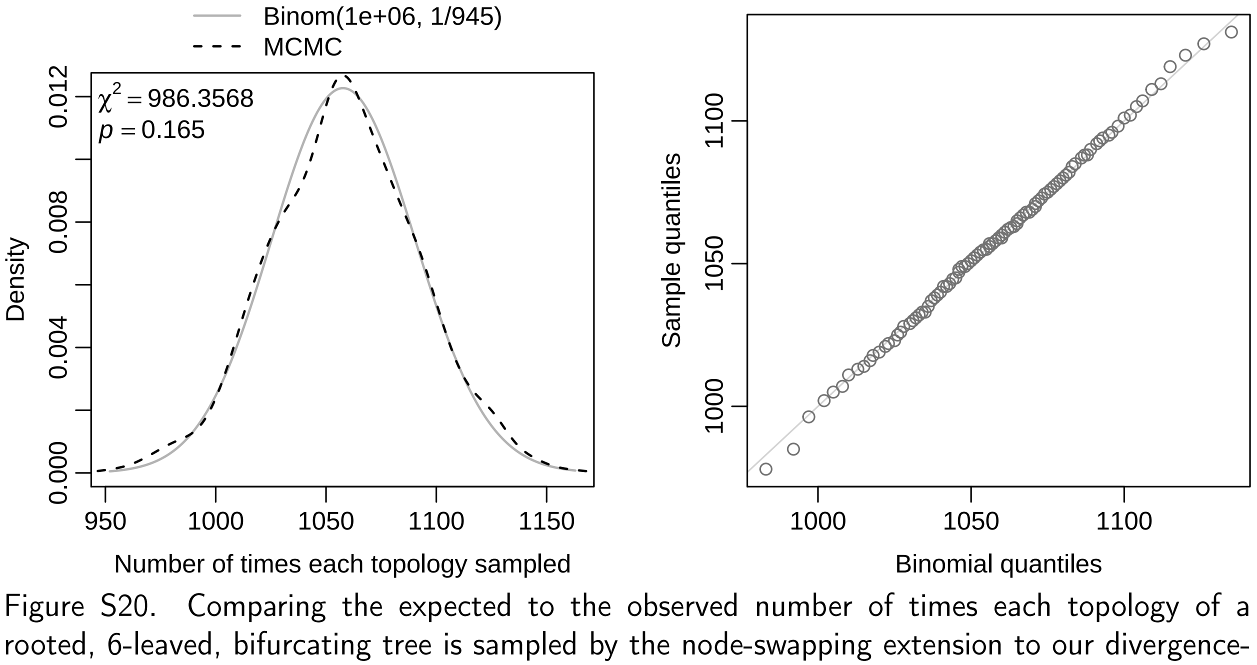

Figure S20. Comparing the expected to the observed number of times each topology of a rooted, 6-leaved, bifurcating tree is sampled by the node-swapping extension to our divergence-time-slide-bump move. Our MCMC sample of 1 million trees closely matched the expected Binomial($n = 1 \times 10^6, p = \frac{1}{945}$) distribution.
